# Supplementary material for: Accurate Recycling of Parental Histones Reproduces the Histone Modification Landscape during DNA Replication
Source: Mol Cell. 2018 Oct 18;72(2):239–249.e5. doi: 10.1016/j.molcel.2018.08.010 (PMC6202308; doi:10.1016/j.molcel.2018.08.010)
Supplement: Document S1. Figures S1–S5 [file mmc1.pdf]

Molecular Cell, Volume *72*

Supplemental Information

Accurate Recycling of Parental Histones Reproduces the Histone Modification Landscape during DNA Replication

Nazaret Reverón-Gómez, Cristina González-Aguilera, Kathleen R. Stewart-Morgan, Nataliya Petryk, Valentin Flury, Simona Graziano, Jens Vilstrup Johansen, Janus Schou Jakobsen, Constance Alabert, and Anja Groth

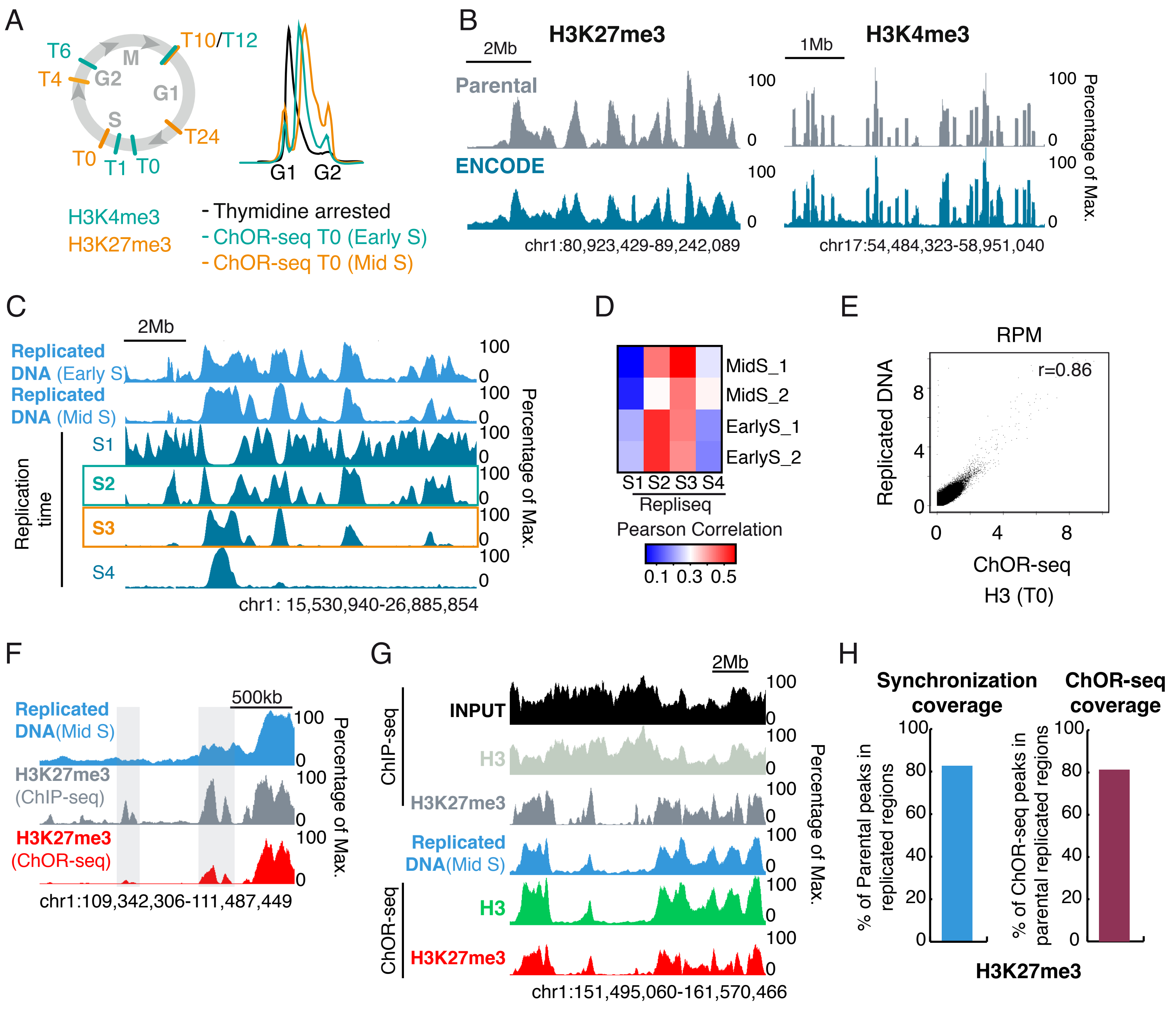

Figure S1. Tracking histone PTM occupancy after DNA replication with ChOR-seq, Related to Figure 1. (A) Outline of time course analysis for H3K4me3 and H3K27me3 ChOR-seq. (left) Experimental set-up. For H3K4me3 (green), cells were pulsed with EdU in early S phase and ChOR-seq samples were harvested immediately (T0, nascent) and 1 (T1), 6 (T6) and 12 (T12) hours later. For H3K27me3 (orange), cells were pulsed with EdU in mid S phase and ChOR-seq samples were harvested immediately (T0, nascent) and 4 (T4), 10 (T10) and 24 (T24) hours later. Cell cycle progression as indicated in the experimental set-up was assessed by FACS analysis of DNA content, as illustrated for T0 (right). **(B)** Profiles of parental H3K27me3 (left) and H3K4me3 (right) ChIP-seq in S phase synchronized cultures (gray) compared to ENCODE profiles from asynchronous Hela S3 cells (blue). Signal is scaled as percentage of maximum at the locus depicted. **(C)** Replicated DNA profiles from samples labelled in early or mid S phase compared with replication timing profiles determined by Repli-seq (ENCODE Project Consortium, 2012). Signal is scaled as percentage of maximum at the locus depicted. **(D)** Heat maps showing the global Pearson correlation between replicated DNA profiles of samples labelled in early or mid S and replication timing profiles determined by Repli-seq (ENCODE Project Consortium, 2012). Correlations are calculated from 500 bp non-overlapping windows and RPM. **(E)** Dot plots comparing signal in replicated DNA and pan-H3 nascent ChOR-seq. Dots represent 500 bp non-overlapping windows within replicated regions. R-values shown are Pearson correlations. **(F)** Profiles of replicated DNA (blue), parental H3K27me3 ChIP-seq (gray) and nascent H3K27me3 ChOR-seq (red). The left highlighted region shows a locus with parental signal but no nascent signal, owing to a low degree of replication (low enrichment of replicated DNA). The right highlighted region shows a locus with high parental signal but moderate nascent signal, owing to it being replicated to a moderate degree. The locus also includes a highly replicated region showing a strong agreement of the nascent signal with the parental. Signal is scaled as percentage of maximum at the locus depicted. **(G)** Parental ChIP-seq and nascent ChOR-seq profiles of pan-H3 and H3K27me3 using biotin-dUTP labelling and omitting spike-in chromatin. Replicated DNA profile is shown in blue. Signal was scaled as percentage of maximum at the locus depicted. **(H)** Bar plots showing the synchronization coverage (left) and ChOR-seq coverage (right) in H3K27me3 datasets from (G). Percentage is calculated from peaks subsetted into 500 bp non-overlapping windows.

Reverón-Gómez et al. Figure S2

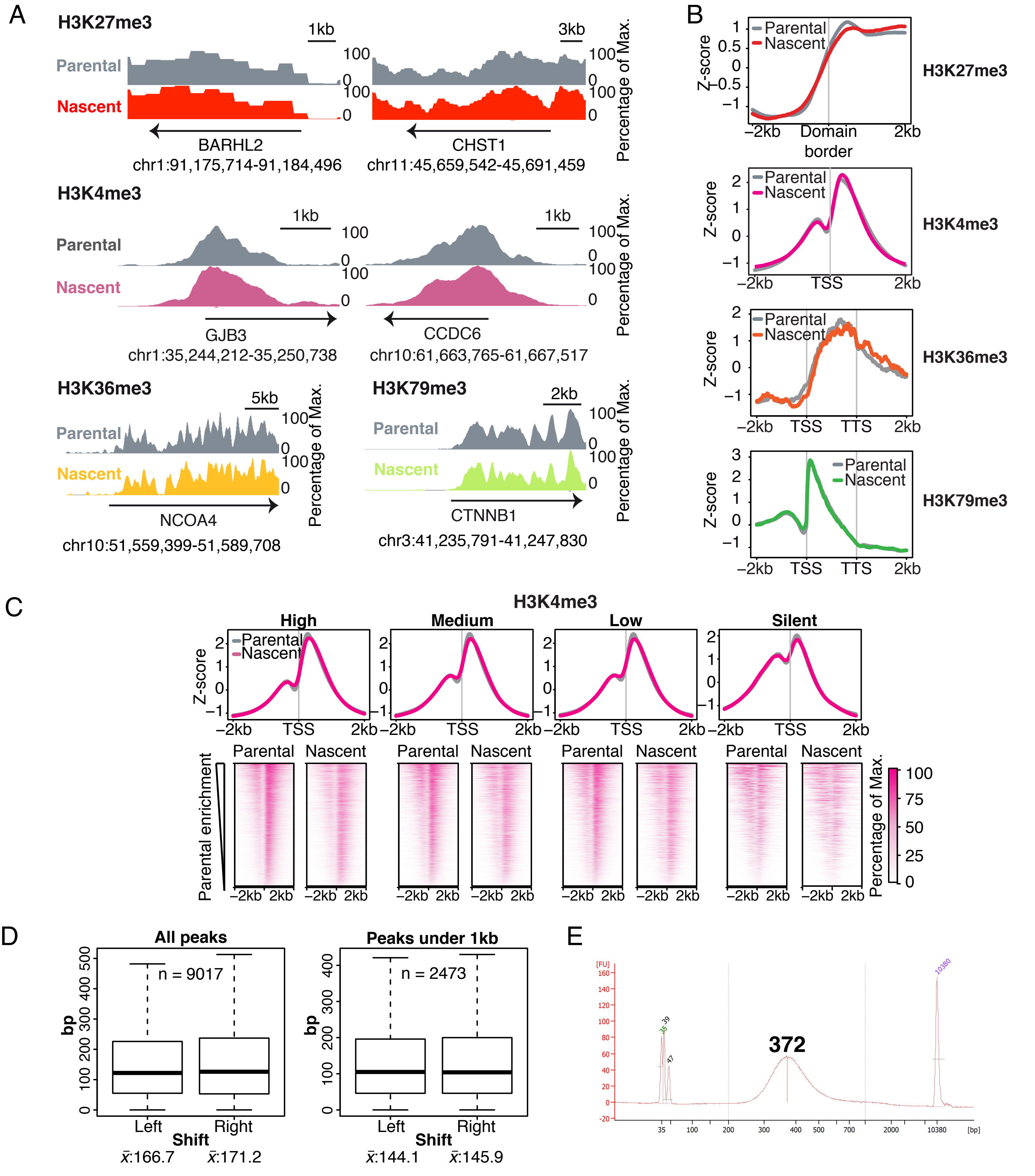

Figure S2. The histone H3 modification landscape is accurately reproduced in active and repressed genomic loci, Related to Figure 2. (A) Profiles of parental ChIP-seqs (gray and nascent H3K27me3 (red, H3K4me3 (pink, H3K36me3 (yellow, and H3K79me3 (green qChOR-seq at high resolution. Signal was scaled as percentage of maximum at the locus depicted. **(B)** Average profiles of parental and nascent H3K27me3, H3K4me3, H3K36me3, and H3K79me3 in replicate 2. H3K27me3 signal is plotted across 4 kb centered on borders of replicated H3K27me3 domains. H3K4me3 signal is plotted across 4 kb centered on replicated TSSs. H3K36me3 and H3K79me3 signals are plotted from 2 kb upstream to 2 kb downstream of replicated open reading frames. All data shown is z-score normalized. **(C)** Average profiles of parental and nascent H3K4me3 at TSSs parsed by expression level. Signal is plotted across 4 kb centered on replicated TSSs. All data shown is z-score normalized. **(D)** Box plots of difference in localization of parental and nascent H3K4me3 peaks (left and right shift) in base pairs for all peaks (left panel) and narrow peaks of less than 1 Kb (right panel). The narrow peaks containing a maximum of 5 nucleosomes provide the best resolution with respect to diffusion. n indicates the number of peaks analyzed. $\bar{x}$ indicates the mean value of the absolute distance in bp between parental and nascent H3K4me3 peaks, note that the median is shown in the box plot. **(E)** Representative Bioanalyzer profile of a H3K4me3 ChOR-seq library. Taking into account the length of adaptors (approximately 120 bp), the average fragment size is 250 bp.

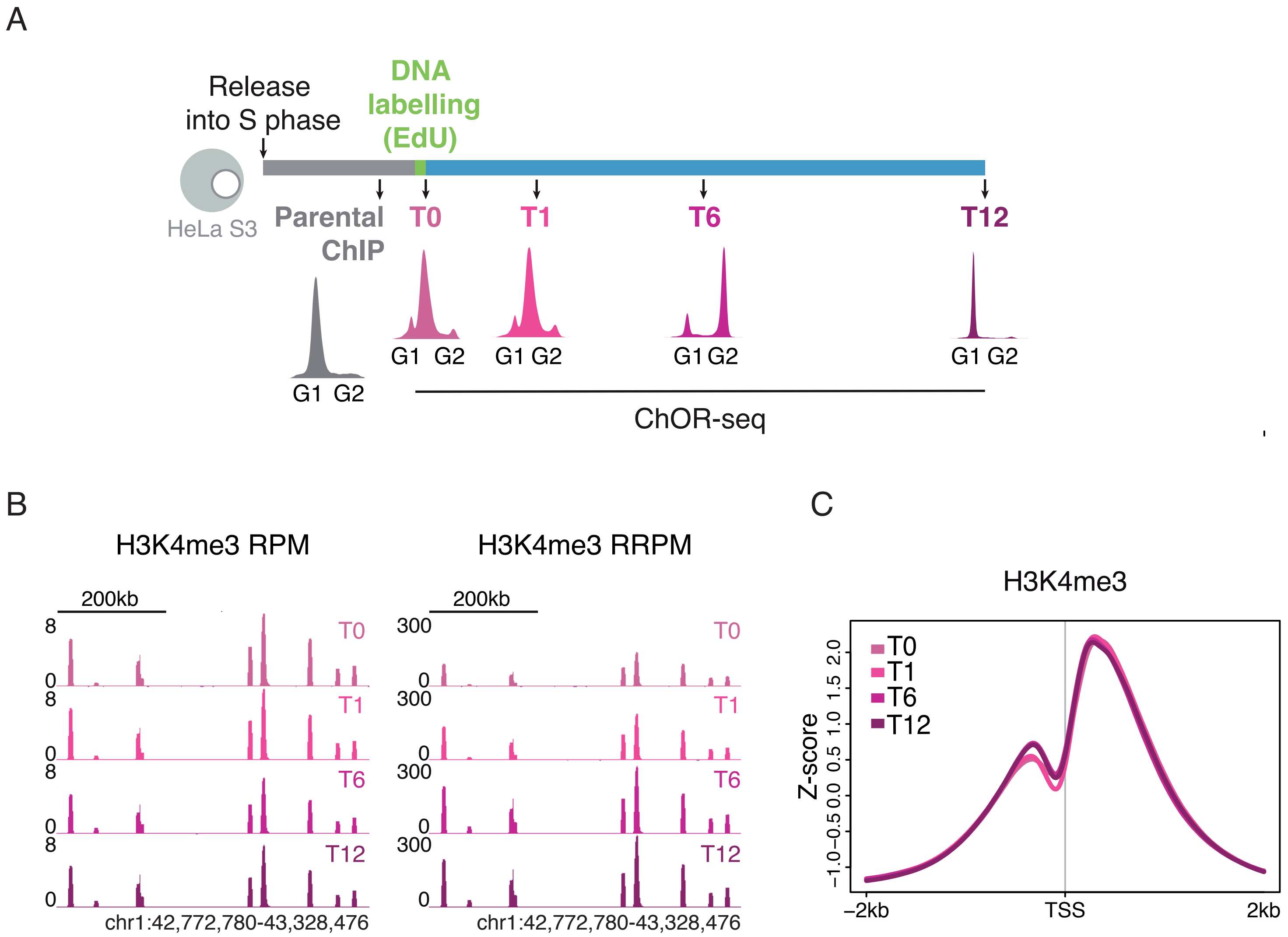

Figure S3. H3K4me3 restoration is complete within 6 hours and fastest in highly expressed promoters, Related to Figure 3. (A) Experimental set-up for H3K4me3 qChOR-seq. Parental chromatin was collected 1 hour before EdU labelling (10 minutes) and samples for qChOR-seq were harvested at the indicated time points post-EdU label. Cell cycle progression was assessed by FACS analysis of DNA content. **(B)** Comparison of H3K4me3 qChOR-seq profiles normalized to reads per million (RPM, left) or reference-adjusted reads per million using exogenous spike-in chromatin (RRPM, right). **(C)** Average profiles of H3K4me3 qChOR-seq. H3K4me3 signal is plotted across 4 kb centered on replicated TSSs. All data shown is z-score normalized.

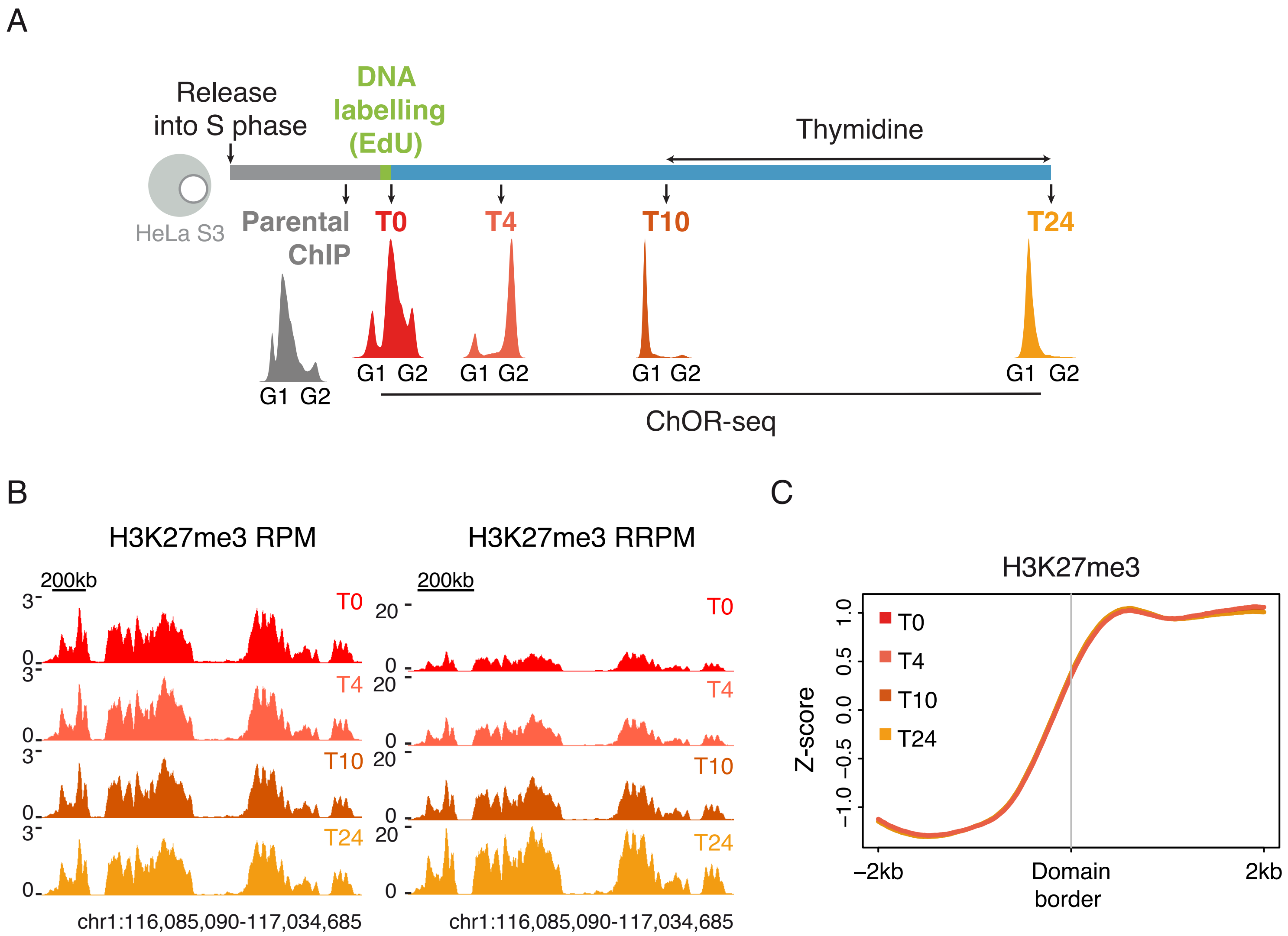

Figure S4. High PRC2 occupancy sites show faster H3K27me3 restoration, Related to Figure 4. (A) Experimental set-up for H3K27me3 qChOR-seq. Parental chromatin was collected 1 hour before EdU labelling (20 minutes) and samples for qChOR-seq were harvested at the indicated time points post-EdU label. Thymidine was added at 12 hours to prevent cells from entering a new round of replication. Cell cycle progression was assessed by FACS analysis of DNA content. **(B)** Comparison of H3K27me3 qChOR-seq profiles normalized to reads per million (RPM, left) or reference-adjusted reads per million using exogenous spike-in chromatin (RRPM, right). **(C)** Average profiles of H3K27me3 qChOR-seq. H3K27me3 signal is plotted across 4 kb centered on borders of replicated H3K27me3 domains. All data shown is z-score normalized.

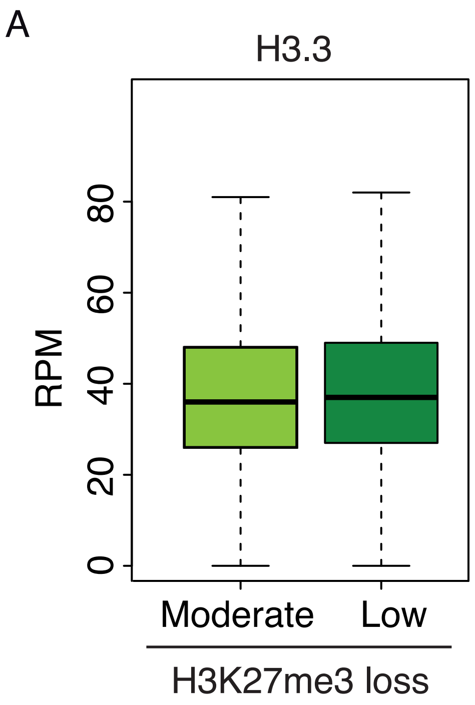

Figure S5. Parental H3K27me3 domains are stable across the cell cycle, Related to Figure 5. (A) Box plots of H3.3 ChIP-seq signal in moderate and low H3K27me3 loss regions. Data is subsetted into 2 kb non-overlapping windows and quantitated using reads per million (RPM). H3.3 data are from (Ray-Gallet et al., 2011).
