# Supplementary material for: Accurate Recycling of Parental Histones Reproduces the Histone Modification Landscape during DNA Replication
Source: Mol Cell. 2018 Oct 18;72(2):239–249.e5. doi: 10.1016/j.molcel.2018.08.010 (PMC6202308; doi:10.1016/j.molcel.2018.08.010)
Supplement: Document S2. Article plus Supplemental Information [file mmc2.pdf]

Molecular Cell

Article

Accurate Recycling of Parental Histones Reproduces the Histone Modification Landscape during DNA Replication

Graphical Abstract

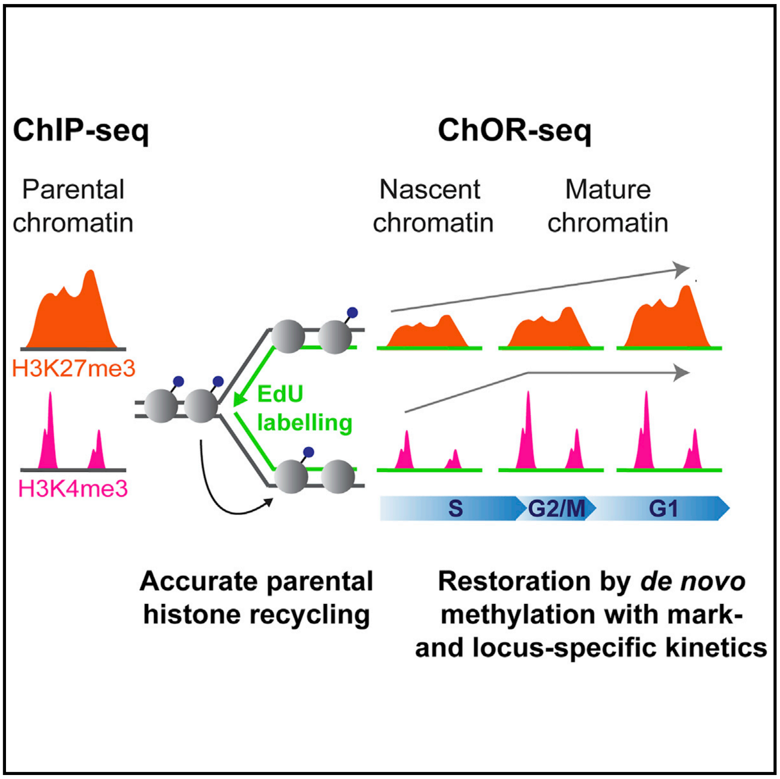

Authors

Nazaret Reverón-Gómez, Cristina González-Aguilera, Kathleen R. Stewart-Morgan, ..., Janus Schou Jakobsen, Constance Alabert, Anja Groth

Correspondence

anja.groth@bric.ku.dk

In Brief

Histone modifications are a core component of the epigenome. Reverón-Gómez et al. develop ChOR-seq to profile histone modifications after DNA replication and find that the genomic localization of modified parental histones is preserved on daughter strands while new histone modification to restore pre-replication levels follows mark- and locus-specific kinetics.

Highlights

- ChOR-seq determines histone PTM occupancy on newly replicated DNA
- Histone PTM positional information is preserved through parental histone recycling
- Parental H3K27me3 domains are stable and inherited to daughter cells
- Restoration of histone PTM levels follows mark- and locus-specific kinetics

Reverón-Gómez et al., 2018, Molecular Cell *72*, 239–249
October 18, 2018 © 2018 The Authors. Published by Elsevier Inc.
https://doi.org/10.1016/j.molcel.2018.08.010

Accurate Recycling of Parental Histones Reproduces the Histone Modification Landscape during DNA Replication

Nazaret Reverón-Gómez,[1,2,6] Cristina González-Aguilera,[1,3,6] Kathleen R. Stewart-Morgan,[1,2,6] Nataliya Petryk,[1,2] Valentin Flury,[1,2] Simona Graziano,[1,2] Jens Vilstrup Johansen,[1] Janus Schou Jakobsen,[1,4] Constance Alabert,[1,5] and Anja Groth[1,2,7,*]

[1]Biotech Research and Innovation Centre (BRIC), University of Copenhagen, Faculty of Health and Medical Sciences, University of Copenhagen, 2200 Copenhagen, Denmark
[2]The Novo Nordisk Center for Protein Research (CPR), University of Copenhagen, Faculty of Health and Medical Sciences, University of Copenhagen, 2200 Copenhagen, Denmark
[3]Present address: Andalusian Centre for Molecular Biology and Regenerative Medicine (CABIMER), CSIC/University of Seville, Seville 41092, Spain
[4]Present address: Symphogen A/S, 2750 Ballerup, Denmark
[5]Present address: Centre for Gene Regulation and Expression, School of Life Sciences, University of Dundee, Dundee DD1 5EH, UK
[6]These authors contributed equally
[7]Lead Contact
*Correspondence: anja.groth@bric.ku.dk
https://doi.org/10.1016/j.molcel.2018.08.010

SUMMARY

Chromatin organization is disrupted genome-wide during DNA replication. On newly synthesized DNA, nucleosomes are assembled from new naive histones and old modified histones. It remains unknown whether the landscape of histone post-translational modifications (PTMs) is faithfully copied during DNA replication or the epigenome is perturbed. Here we develop chromatin occupancy after replication (ChOR-seq) to determine histone PTM occupancy immediately after DNA replication and across the cell cycle. We show that H3K4me3, H3K36me3, H3K79me3, and H3K27me3 positional information is reproduced with high accuracy on newly synthesized DNA through histone recycling. Quantitative ChOR-seq reveals that *de novo* methylation to restore H3K4me3 and H3K27me3 levels occurs across the cell cycle with mark- and locus-specific kinetics. Collectively, this demonstrates that accurate parental histone recycling preserves positional information and allows PTM transmission to daughter cells while modification of new histones gives rise to complex epigenome fluctuations across the cell cycle that could underlie cell-to-cell heterogeneity.

INTRODUCTION

The organization of eukaryotic genomes into chromatin influences all DNA-based processes, including gene expression and DNA repair. Chromatin organization is particularly important for establishing and maintaining cell-type-specific transcriptional programs and thus underlies epigenetic cell memory (Allis and Jenuwein, 2016; Halley-Stott and Gurdon, 2013). However, the basic mechanisms that ensure propagation of chromatin states during DNA replication and across cell division remain unclear (Alabert and Groth, 2012; Allis and Jenuwein, 2016; Almouzni and Cedar, 2016).

The nucleosome is the basic unit of chromatin, in which 146 base pairs of DNA are wrapped around a histone core composed of a central histone H3-H4 tetramer flanked by two histone H2A-H2B dimers. Histones are decorated with a large variety of post-translational modifications (PTMs) that contribute to the establishment and maintenance of active and repressed chromatin states (Patel and Wang, 2013). Many of these regulatory modifications are found on histone H3. Histone H3 lysine 4 tri-methylation (H3K4me3), histone H3 lysine 36 tri-methylation (H3K36me3), and histone H3 lysine 79 tri-methylation (H3K79me3) mark active chromatin, with H3K4me3 enriched in promoter regions and H3K36me3 and H3K79me3 enriched in gene bodies (Rando, 2007). Conversely, tri-methylation of histone H3 lysine 27 (H3K27me3) demarcates larger, transcriptionally silent domains (Schuettengruber et al., 2017).

Histone modifications associated with both transcriptional silencing and activation are proposed to play a central role in epigenetic cell memory (Allis and Jenuwein, 2016; Campos et al., 2014; Halley-Stott and Gurdon, 2013), implying that histone-based information must be transferred to daughter cells during mitotic cell division. However, the process of DNA replication is disruptive and leads to the disassembly of nucleosomes into H3-H4 tetramers and H2A-H2B dimers (Jackson, 1987, 1988, 1990; Xu et al., 2010). Electron microscopy and *in vitro* replication of simian virus 40 (SV40) mini-chromosomes have shown that 1–2 parental nucleosomes are destabilized ahead of the replication fork (Gasser et al., 1996; McKnight and Miller, 1977) and that histones are released from DNA, but kept in close

This is an open access article under the CC BY license (http://creativecommons.org/licenses/by/4.0/).

proximity, during fork passage (Gruss et al., 1993; Madamba et al., 2017). Shortly after fork passage, nucleosome density is restored on the two daughter DNA strands through a combination of re-deposition (recycling) of old histones and incorporation of newly synthesized histones (Alabert and Groth, 2012; Almouzni and Cedar, 2016; Annunziato, 2015). Recent nucleosome mapping experiments in *Drosophila* S2 cells and yeast have revealed that nucleosome occupancy is increased around active promoter and enhancer regions shortly after DNA replication (Fennessy and Owen-Hughes, 2016; Ramachandran and Henikoff, 2016; Vasseur et al., 2016), but it is unclear whether this reflects new histone incorporation or dispersal of positioned parental nucleosomes.

A central question in epigenetics is therefore how genome-wide chromatin disruption during DNA replication might be compatible with inheritance of genomic histone modification patterns to daughter cells. Quantitative proteomic analyses of new and old histones in human cells have shown that old and new histones (H3, H4, H2A, and H2B) are mixed in a 1:1 ratio on newly replicated DNA (Alabert et al., 2015) and that old histone H3-H4 are recycled with their modifications (Alabert et al., 2015; Pesavento et al., 2008; Scharf et al., 2009; Xu et al., 2011; Zee et al., 2012). Old histone H3-H4 dimers do not mix with new ones (Jackson, 1987, 1990; Xu et al., 2010), arguing that intact, old H3-H4 tetramers with their PTM information are transferred onto newly synthesized DNA. However, it is not known how precisely old histones are re-incorporated on the new daughter DNA strands relative to their former genomic position or whether histones and their associated marks are dispersed during DNA replication. This is particularly important because modified parental histones may direct modifying enzymes toward new histones in their vicinity (Audergon et al., 2015; Brown et al., 2017; Patel and Wang, 2013; Ragunathan et al., 2015) and allosteric regulation of the PRC2 complex by H3K27me3 facilitates a positive feedforward loop (Jiao and Liu, 2015; Margueron et al., 2009). Mathematical modeling has estimated that old histones are reincorporated within 400 bp of their original genomic location in yeast (Radman-Livaja et al., 2011). However, the re-deposition of parental histones has not been tracked directly.

To understand how the PTM landscape is duplicated during DNA replication, it is necessary to elucidate where and when modified histones are deposited within a given genomic locus post replication. We have developed a technology to analyze chromatin occupancy after DNA replication by next-generation sequencing, termed ChOR-seq. ChOR-seq can track the occupancy of proteins and histone PTMs after replication fork passage genome-wide. Given that newly synthesized histones are devoid of tri-methylation marks at the time of deposition (Alabert et al., 2015; Bar-Ziv et al., 2016; Jasencakova et al., 2010; Loyola et al., 2006), ChOR-seq provides a means to track recycling of old modified histones and to directly measure potential replication-dependent displacement of pre-existing histone PTMs. Using ChOR-seq to track H3K4me3, H3K36me3, H3K79me3, and H3K27me3, we find that PTM occupancy patterns are reproduced on newly replicated DNA with high accuracy in both repressed and active genomic regions, demonstrating that the positional information of histone marks is faithfully inherited to daughter strands during DNA replication. We then track restoration of H3K4me3 and H3K27me3 levels by quantitative ChOR-seq time course analysis and find that *de novo* histone methylation after DNA replication increases the level of the marks within regions already demarcated by modified parental histones. Notably, we find that restoration of histone PTM levels follows mark- and locus-specific kinetics, arguing that the epigenome is undergoing complex changes across the cell cycle that could underlie cell-to-cell heterogeneity.

RESULTS

ChOR-Seq Tracks Protein and PTM Occupancy on Replicated DNA

ChOR-seq is based on short (10–20 min) pulse labeling of replicated DNA with a nucleotide analog (EdU) followed by sequential chromatin immunoprecipitation (ChIP) of a specific histone PTM. Labeled DNA is then biotinylated via Click-IT and isolated by biotin-streptavidin pull-down prior to analysis by next-generation sequencing (Figure 1A). To investigate histone modification patterns after DNA replication, we first performed ChOR-seq experiments for H3K27me3 and H3K4me3. Since H3K27me3 and H3K4me3 are markers of repressed and active chromatin, respectively, this approach allowed us to assess the ChOR-seq method in distinct regions of the genome. We also performed ChOR-seq of pan-histone H3 to track overall nucleosome occupancy. To inform on pre-replication histone PTM position, we used S phase synchronized HeLa S3 and carried out standard ChIP-seq of H3K4me3 and H3K27me3 in total chromatin prior to DNA labeling (parental ChIP) (Figure 1B; Figure S1A). These H3K4me3 and H3K27me3 enrichment profiles from S-phase-synchronized cells were largely identical to genome-wide maps of H3K4me3 and H3K27me3 in asynchronous HeLa S3 cells available from ENCODE (Bernstein et al., 2005) (Figure S1B), confirming that parental ChIP-seq is a suitable baseline for assessing our ChOR-seq data. Our synchronization setup also allowed us to verify the specific isolation of replicated DNA by comparison to replication timing data available for HeLa S3 cells (Figures S1C and S1D) (ENCODE Project Consortium, 2012). To optimize coverage of transcriptionally active and repressed loci, we labeled replicating DNA in early S phase and mid S phase, respectively, corresponding to the replication timing of these regions (Comoglio and Paro, 2014; Julienne et al., 2013; Pope et al., 2014). We harvested samples for ChOR-seq immediately after EdU pulse labeling (nascent chromatin) and at selected later time points to track chromatin maturation (mature chromatin) (Figure 1B; Figure S1A). Finally, to allow later quantitative analyses of histone PTM levels during maturation, we spiked in EdU-labeled chromatin from *Drosophila* S2 cells (Figure 1A) (Bonhoure et al., 2014).

ChOR-seq profiles of pan-H3 showed a high correlation (r = 0.86) with replicated DNA profiles (EdU pull-downs) (Figure 1C; Figure S1E). This was expected due to the rapid restoration of nucleosome occupancy on newly replicated DNA (Annunziato, 2015; McKnight and Miller, 1977) and provided confirmation that ChOR-seq determines occupancy specifically on replicated DNA. Further confirmations of ChOR-seq specificity were instances in which regions of parental ChIP-seq enrichment lacked ChOR-seq signal owing to insufficient

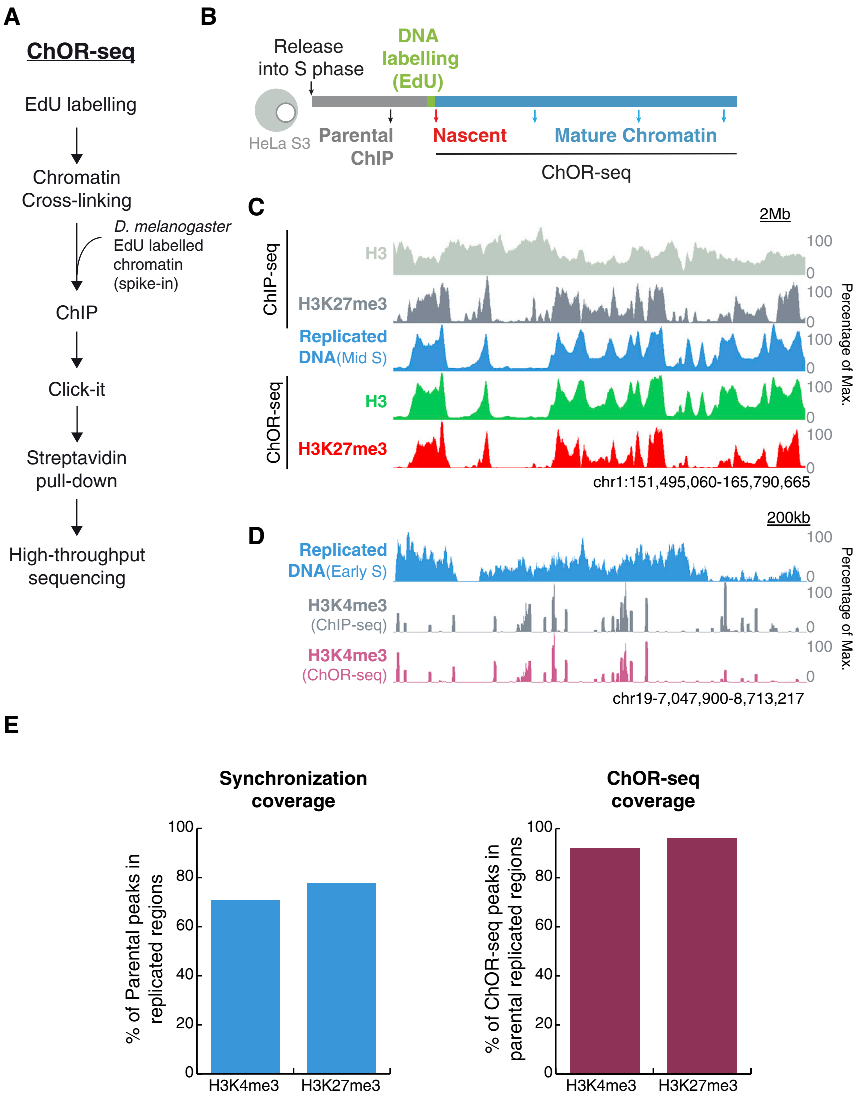

Figure 1. Tracking Histone PTM Occupancy after DNA Replication with ChOR-Seq

(A) Overview of the ChOR-seq protocol.

(B) Experimental setup. HeLa S3 cells were released into S phase from a thymidine block. Parental and nascent chromatin were collected 1 hr before or immediately after EdU labeling, respectively. The EdU label was then chased and mature chromatin harvested at selected time points along the cell cycle.

(C and D) Parental ChIP-seq and nascent ChOR-seq profiles of pan-H3 and H3K27me3 (C) and H3K4me3 (D). Replicated DNA profiles are shown in blue. Signal is scaled as percentage of maximum at the locus depicted.

(E) Bar plots showing the synchronization coverage (left) and ChOR-seq coverage (right) in the H3K4me3 and H3K27me3 datasets. Percentage is calculated from peaks subsetted into 500 bp non-overlapping windows.

See also Figure S1.

replication of the locus at the time of EdU labeling (Figures 1C and 1D; Figure S1F). Our synchronization approach to ChOR-seq captured approximately 70% and 77% of the parental H3K4me3- and H3K27me3-enriched regions, respectively (Figure 1E). Importantly, 94% of H3K27me3 and 92% of H3K4me3 loci in replicated regions were also identified by ChOR-seq (Figure 1E). This was also true when newly replicated DNA was labeled directly by biotin-dUTP instead of EdU coupled with Click-IT chemistry, and the spiked-in *Drosophila* chromatin was omitted (Figures S1G and S1H). We were therefore confident that ChOR-seq was a robust and sensitive method that could directly assess histone PTM occupancy on replicated DNA genome-wide.

The Histone Modification Landscape Is Accurately Reproduced on Newly Synthesized DNA

To address how accurately histone PTM profiles are copied during replication, we compared occupancy patterns of four modifications—H3K27me3, H3K4me3, H3K36me3, and H3K79me3—in pre-replicative and nascent chromatin by parental ChIP-seq and ChOR-seq, respectively. Locally, we observed that histone modification patterns were preserved during replication (Figure 2A; Figure S2A). Plotting averaged signal over sites of expected enrichment for each mark confirmed that this position preservation occurred genome-wide (Figure 2B; Figure S2B). Heatmaps of signal over expected sites of enrichment revealed that this held true for all levels of PTM enrichment (Figure 2C). Parsing H3K4me3 regions by expression level also showed that the accuracy of parental histone deposition was unaffected by parental PTM levels (Figure S2C). Blurring of PTM occupancy at sites of expected enrichment would have indicated dispersal of parental histones during DNA replication. The average profiles of parental and nascent PTM signals did not show any indication of blurring or replication-dependent dispersal of histone PTMs. We further determined the mean difference in localization between nascent and parental H3K4me3 peaks at individual loci to be approximately 170 bp (Figure S2D). This is below the resolution of our ChOR-seq analysis given by an average DNA fragment size of 250 bp (Figure S2E). We thus conclude that parental histones decorated with PTMs are re-incorporated into replicated DNA within 250 bp of their pre-replication position.

H3K4me3 Is Restored within 6 hr Post Replication

ChOR-seq analysis of nascent chromatin showed that histone H3K4me3 occupancy patterns were accurately reproduced on newly replicated DNA, but it remained unclear whether the H3K4me3 landscape was, in fact, fully restored or chromatin maturation would be required for modification of new histones. This was particularly important to address as

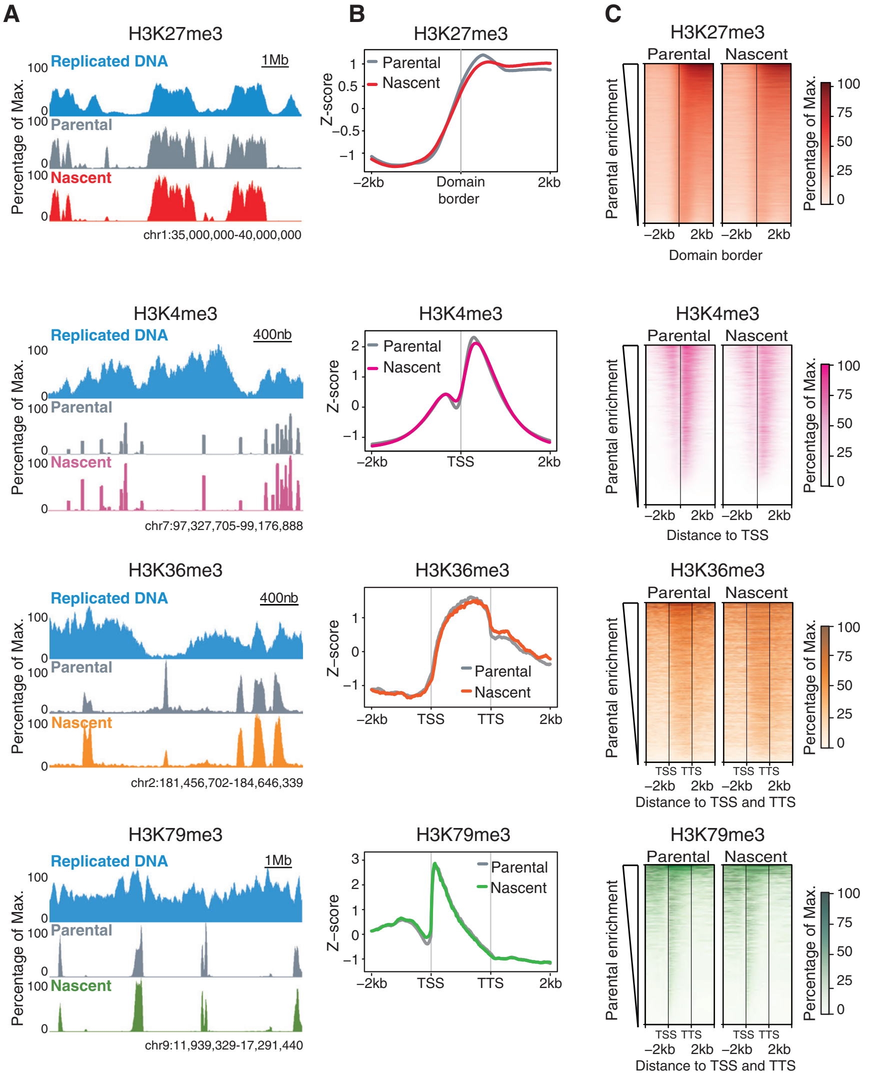

Figure 2. The Histone H3 PTM Landscape Is Accurately Reproduced upon Replication of Active and Repressed Genomic Loci

(A) Histone PTM profiles from ChIP-seq (parental) and ChOR-seq (nascent) of H3K27me3, H3K4me3, H3K36me3, and H3K79me3. Replicated DNA profiles are shown in blue. Signal is scaled as percentage of maximum at the locus depicted.

(B) Average profiles of parental and nascent H3K27me3, H3K4me3, H3K36me3, and H3K79me3. H3K27me3 signal is plotted across 4 kb centered on borders of replicated H3K27me3 domains. H3K4me3 signal is plotted across 4 kb centered on replicated TSSs. H3K36me3 and H3K79me3 signal is plotted from 2 kb upstream to 2 kb downstream of replicated open reading frames. All data shown is *Z* score normalized.

(C) Heatmaps of parental and nascent H3K27me3, H3K4me3, H3K36me3, and H3K79me3 signal across the regions described in (B). Color intensity represents percentage of maximum levels set separately for parental and nascent samples.

See also Figure S2.

H3K4me3 cell-cycle dynamics have not been resolved by mass spectrometry due to technical limitations. Resolving H3K4me3 restoration kinetics is, however, amenable to quantitative ChOR-seq (qChOR-seq), which takes advantage of a *Drosophila* chromatin spike-in to normalize read counts, revealing quantitative differences in signal between samples that are lost with conventional data processing methods. We therefore carried out H3K4me3 qChOR-seq on nascent chromatin and on mature chromatin harvested 1 hr later, with cells still in S phase (T1); 6 hr later, when cells reached G2/M but had not passed through mitosis (T6); and 12 hr later, when cells had passed through mitosis and were in G1 of the next cell cycle (T12) (Figure 3A; Figure S3A). While the raw ChOR-seq signals (RPM) were highly similar (Figure S3B), normalization using spiked-in EdU-labeled *Drosophila* chromatin (RRPM) revealed a substantial accumulation of H3K4me3 during the first 6 hr of chromatin maturation (Figure 3B; Figure S3B).

Genome-wide, we observed marked gains in H3K4me3 signal between T0 and T1 and T1 and T6 but no further increase between T6 and T12 (Figures 3C and 3D). Notably, this gain of H3K4me3 occurred within the H3K4me3 regions already present in nascent chromatin and did not lead to expansion of H3K4me3 peaks over time (Figure 3D; Figure S3C). Thus, while the genomic location of H3K4me3 is established at the time of DNA replication, restoration of H3K4me3 levels is uncoupled from DNA replication similar to other histone di- and tri-methylation marks (Alabert et al., 2015). However, establishment of H3K4me3 on new histones is largely complete by G2, prior to mitosis, in contrast to repressive marks like H3K27me3 and H3K9me3 that are restored primarily in G1 of the next cell cycle (Alabert et al., 2015).

Expression and CpG Content Predict H3K4me3 Restoration Kinetics

The advantage of qChOR-seq is that it provides both quantitative and positional information about PTM signal. We therefore next asked whether H3K4me3 restoration occurs with different kinetics in different parts of the genome. To address this, we compared qChOR-seq signal across our time course and considered a locus restored when it reached H3K4me3 levels close to that observed at the 12-hr time point (Figure 3E; STAR Methods). This revealed that approximately 50% of H3K4me3 regions are restored within 1 hr of replication (R1), and the remaining 50% are restored within 6 hr of replication (R6) (Figure 3E). H3K4me3 data from ENCODE, produced in asynchronous HeLa cells, showed higher signal over R1 regions (Figure 3F). Since signal at H3K4me3 peaks positively correlate

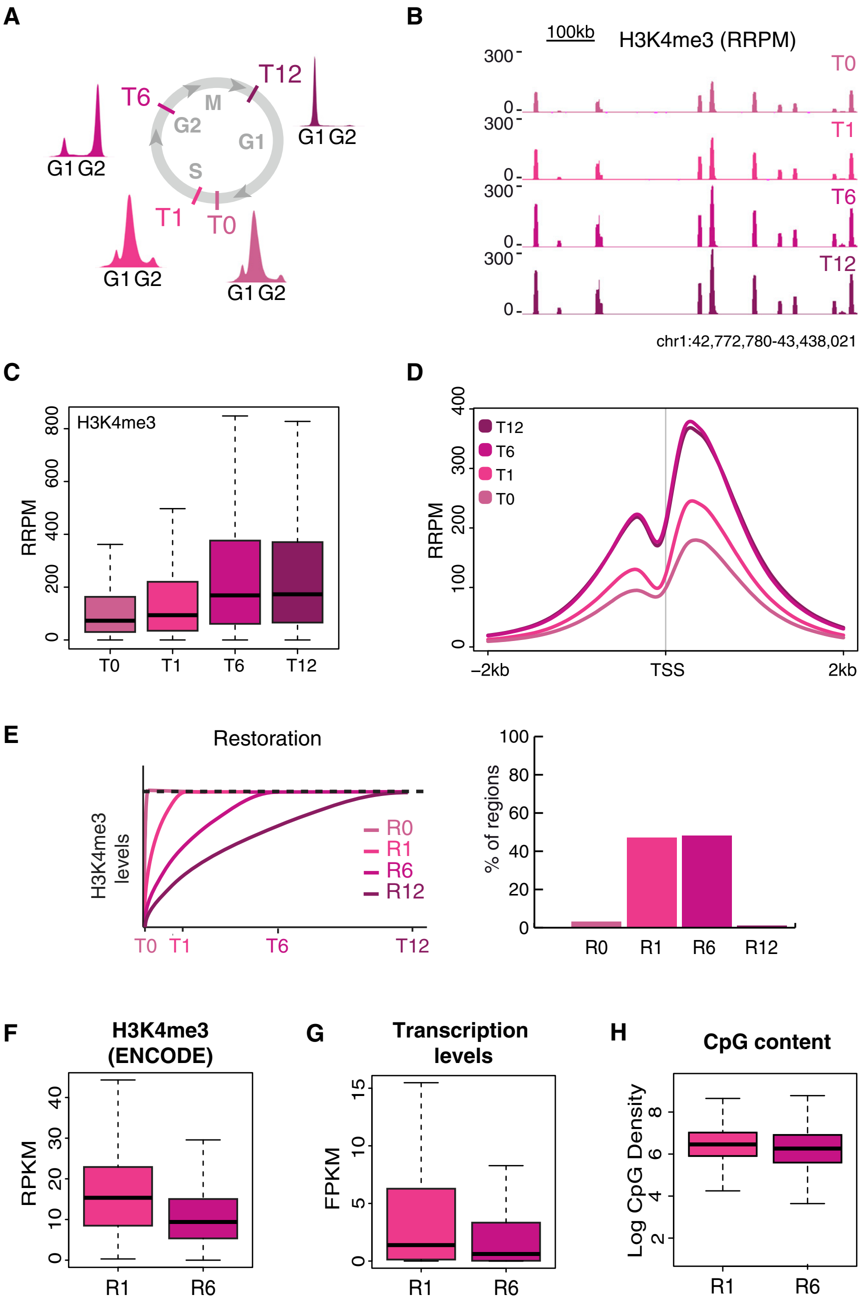

Figure 3. H3K4me3 Restoration Is Complete within 6 hr with Fastest Kinetics in Highly Expressed Promoters

(A) Outline of H3K4me3 qChOR-seq time course analysis. Cell cycle progression was monitored by FACS analysis of DNA content.

(B) Comparison of H3K4me3 nascent and mature qChOR-seq profiles.

(C) Boxplot of H3K4me3 qChOR-seq signal in replicated parental peaks subsetted into 25 bp non-overlapping windows.

(D) Average profiles of H3K4me3 qChOR-seq signal across 4 kb centered on replicated TSSs.

In (B)–(D), signal is quantitated using reference-adjusted reads per million (RRPM).

(E) Left: scheme of strategy used to parse H3K4me3-enriched regions by restoration kinetics. Regions were defined as R0, R1, R6, or R12 based on the time point at which R12 H3K4me3 levels were reached. Right: bar chart of the proportion of H3K4me3-enriched regions in each restoration category. Regions are defined as 500 bp non-overlapping windows in replicated parental peaks.

(F) Boxplot of ENCODE H3K4me3 signal in R1 and R6 regions. Signal is quantitated using reads per kilobase per million (RPKM).

(G) Boxplot of RNA-seq signal over genes associated with R1 and R6 promoters. RNA-seq data are from Mortazavi et al. (2008). Signal is quantitated using fragments per kilobase per million (FPKM).

(H) Boxplot showing the CpG densities of CpG islands overlapping R1 and R6 regions. CpG content data are from Illingworth et al. (2010).

See also Figure S3.

with both the expression level of associated genes and the CpG density of the underlying DNA, we next compared R1 and R6 regions with respect to these two characteristics. Consistent with our predictions based on the H3K4me3 signal from ENCODE, transcription start sites (TSSs) within R1 regions were more highly expressed (Figure 3G), and R1 regions were more CpG dense (Figure 3H) than those in the R6 restoration category. This implicates both transcription and DNA sequence content as important determinants of H3K4me3 restoration kinetics. Taken together, our qChOR-seq time course has both defined H3K4me3 restoration kinetics globally and revealed important, site-specific differences in restoration rates, complementing existing knowledge about H3K4me3 biology while adding crucial and novel insights into the propagation of this mark.

High PRC2 Occupancy Sites Show Faster H3K27me3 Restoration

We have previously defined the restoration kinetics of H3K27me3 at the global level using quantitative mass spectrometry (Alabert et al., 2015). Bulk mass spectrometry methods, however, are unable to detect site-specific differences in PTM restoration. Therefore, to address how the H3K27me3 landscape develops across the cell cycle and reveal whether particular genomic loci restore with faster kinetics than bulk H3K27me3, we carried out H3K27me3 qChOR-seq time course analyses. We included restoration times of 4 hr, when cells were in G2 phase; 10 hr, corresponding to early G1 phase; and 24 hr, where we arrested cells at the G1/S transition to avoid re-replication of the domains (Figure 4A; Figure S4A). Applying spike-in normalization to allow quantitative comparison (Figure S4B) revealed that H3K27me3 qChOR-seq signal gradually accumulated across all time points, with the major increase taking place after mitosis in daughter cells (Figure 4B), corroborating the

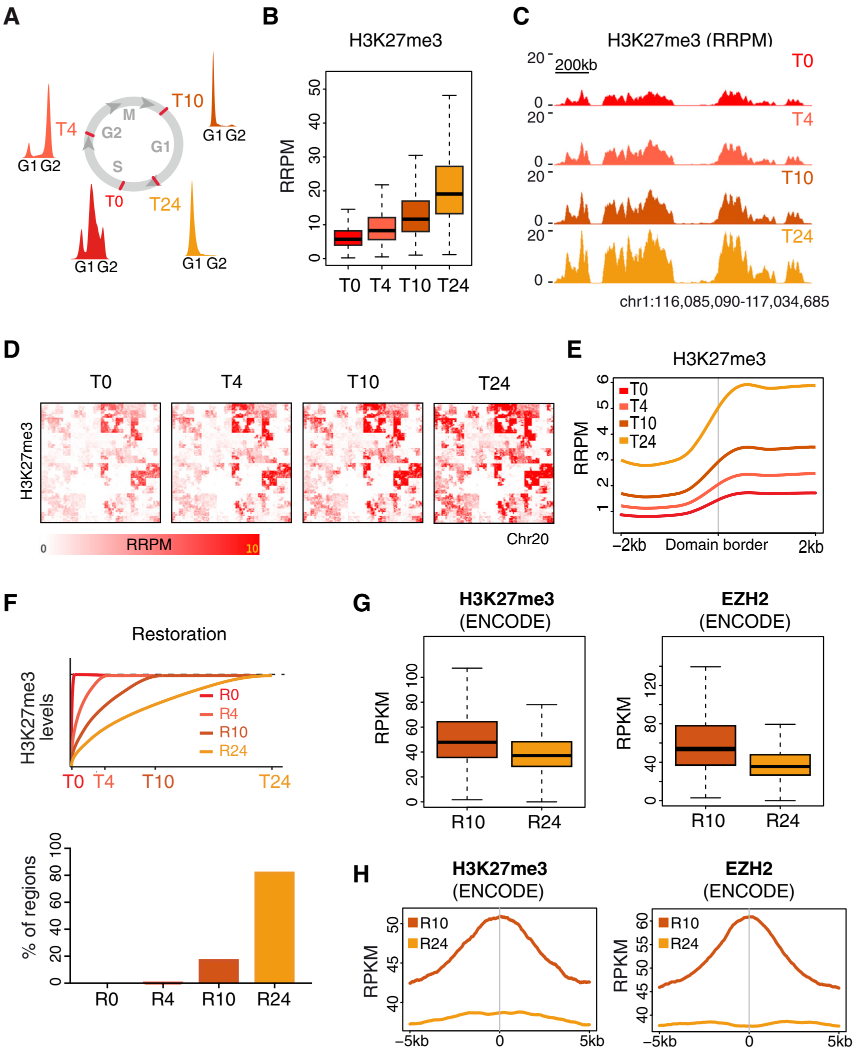

Figure 4. High PRC2 Occupancy Sites Show Faster H3K27me3 Restoration

(A) Outline of H3K27me3 qChOR-seq time course analysis. Cell cycle progression was monitored by FACS analysis of DNA content.

(B) Boxplots of H3K27me3 qChOR-seq signal in replicated parental peaks subsetted into 2 kb non-overlapping windows.

(C) Comparison of H3K27me3 nascent and mature qChOR-seq profiles.

(D) Hilbert curves of H3K27me3 qChOR-seq signal over chromosome 20 at the indicated time points. Colored areas reflect the size and signal of H3K27me3-enriched domains.

(E) Average profiles of H3K27me3 qChOR-seq signal across 4 kb centered on the border of replicated H3K27me3 domains.

In (B)–(E), signal is quantitated using reference-adjusted reads per million (RRPM).

(F) Top: scheme of strategy used to parse H3K27me3-enriched regions by restoration kinetics. Regions were defined as R0, R4, R10, or R24 based on the time point at which R24 H3K27me3 levels were reached. Bottom: bar chart of the proportion of H3K27me3-enriched regions in each restoration category. Regions are defined as 2 kb non-overlapping windows in replicated parental peaks.

(G) Boxplots of ENCODE H3K27me3 signal (left) and ENCODE EZH2 signal (right) in R10 and R24 regions. Signal is quantitated using reads per kilobase per million (RPKM).

(H) Average profiles of ENCODE H3K27me3 signal (left) and ENCODE EZH2 signal (right) across 10 kb centered on R10 and R24 regions.

See also Figure S4.

previous results from quantitative mass spectrometry (Alabert et al., 2015). Importantly, this gradual increase in H3K27me3 levels was evident both at the level of individual domains (Figure 4C) and at the level of whole chromosomes (Figure 4D), with the gain in H3K27me3 being restricted to regions already demarcated by H3K27me3 in nascent chromatin. Looking with higher resolution, we observed that H3K27me3 domain borders were faithfully demarcated at all time points (Figure 4E; Figure S4C). Chromatin restoration thus increases H3K27me3 levels within domains without changing their width, comparable to how H3K4me3-enriched regions were restored.

To identify genomic loci with differential restoration rates, we parsed all replicated H3K27me3 regions according to when the maximal H3K27me3 level was reached. We considered a region restored when reaching a level close to that observed at the 24-hr time point (Figure 4F; STAR Methods). With this definition, we found that about 80% of the analyzed H3K27me3 regions were restored with very slow kinetics, taking up to 24 hr to reach their final level (R24; Figure 4F). We also identified a substantial number of sites restored within 10 hr (R10; Figure 4F), but almost none showing restoration prior to mitosis (R0 and R4; Figure 4F). These results support a model in which old, recycled H3K27me3-marked histones contribute significantly to the chromatin landscape transmitted to daughter cells, while modification of new histones replenishes H3K27me3 levels mainly after cell division. Comparison with H3K27me3 and EZH2 ENCODE data revealed that the faster restoring sites, on average, had higher H3K27me3 levels and EZH2 occupancy (Figure 4G). Further, these regions corresponded to peaks within ENCODE H3K27me3 and EZH2 domains, in contrast to the slowest restoring regions (Figure 4H). Our qChOR-seq analyses therefore demonstrate that H3K27me3 restoration kinetics are locus specific, with high PRC2 occupancy promoting the most efficient H3K27me3 restoration and border regions restoring more slowly.

Parental H3K27me3 Domains Are Stable across the Cell Cycle

Our qChOR-seq analyses in unperturbed systems accurately revealed PTM restoration dynamics genome-wide but left open the question of PTM domain stability in the absence of a restoration mechanism. New histones are deposited largely without methylation marks, including H3K27 methylation (Alabert et al.,

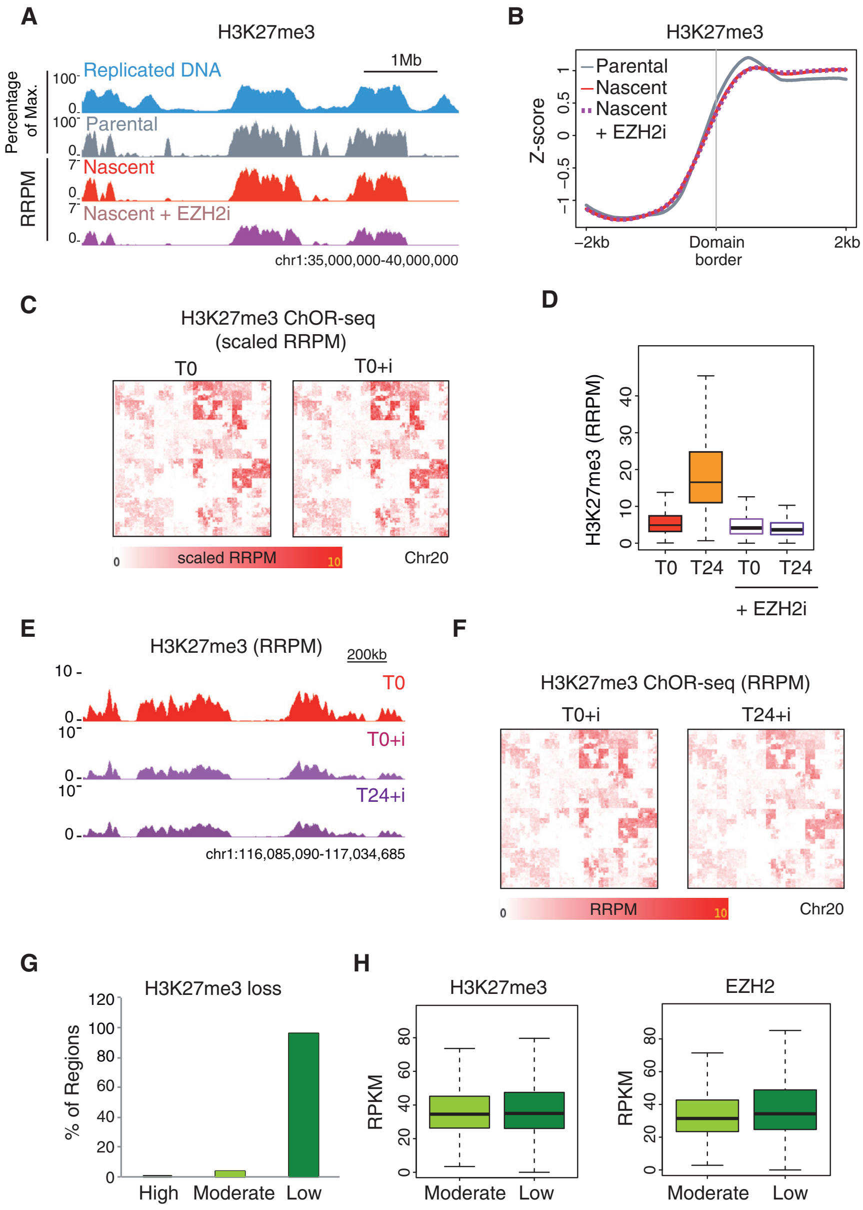

Figure 5. Parental H3K27me3 Domains Are Stable across the Cell Cycle

(A) Profiles of parental H3K27me3 ChIP-seq (gray) and nascent H3K27me3 qChOR-seq in the absence (red) or presence (purple) of EZH2 inhibitor. Replicated DNA is shown in blue. Replicated DNA and parental ChIP-seq signal is scaled as a percentage of maximum at the locus depicted; nascent qChOR-seq signal is quantitated using reference-adjusted reads per million (RRPM).

(B) Average profiles of parental H3K27me3 ChIP-seq (gray) nascent H3K27me3 qChOR-seq signal in the absence (red) or presence (purple dashes) of EZH2 inhibitor. Signal is shown across 4 kb centered on the border of replicated H3K27me3 domains and *Z* score normalized.

(C) Hilbert curves of nascent (T0) H3K27me3 qChOR-seq signal over chromosome 20 in the absence or presence of EZH2 inhibitor. Scaled RRPM values are shown to compare the occupancy landscape (not absolute intensities).

(D) Boxplots of H3K27me3 qChOR-seq signal at T0 and T24 and in the absence or presence of EZH2 inhibitor. Signal is calculated from 2 kb non-overlapping windows in replicated parental peaks.

(E) Profiles of H3K27me3 qChOR-seq at T0 (red) and at T0 and T24 in the presence of EZH2 inhibitor (light and dark purple, respectively).

(F) Hilbert curves of T0 and T24 H3K27me3 qChOR-seq signal over chromosome 20 in the presence of EZH2 inhibitor.

In (D)–(F), signal is quantitated using RRPM.

(G) Bar chart showing the proportion of H3K27me3 regions that exhibit high, moderate, and low qChOR-seq signal loss in the presence of EZH2 inhibitor. Regions were defined as high, moderate, or low loss by comparing T0 and T24 qChOR-seq signal in the presence of EZH2 inhibitor.

(H) Boxplots of ENCODE H3K27me3 signal (left) and ENCODE EZH2 signal (right) in moderate and low loss regions. Signal is quantitated using reads per kilobase per million (RPKM).

See also Figure S5.

2015; Jasencakova et al., 2010; Loyola et al., 2006). H3K27me3, which spans large domains and is catalyzed by a single methyltransferase, EZH2, was therefore an ideal mark to investigate this question. To address the relative contributions of recycled parental histone H3K27me3 and *de novo* histone H3 K27 trimethylation to the inheritance of H3K27me3 to daughter cells, we therefore performed H3K27me3 qChOR-seq analysis in the presence of an inhibitor of EZH2 (Comet et al., 2016; Højfeldt et al., 2018; Knutson et al., 2013) to block new tri-methylation of H3K27 on new and old histones.

We added the EZH2 inhibitor to cells shortly before EdU labeling and performed qChOR-seq to inform on the inheritance of parental histones carrying H3K27me3 both immediately following DNA replication (nascent, T0) and across mitosis to daughter cells (24 hr post EdU labeling, T24). H3K27me3 nascent qChOR-seq revealed that the H3K27me3 occupancy patterns in nascent chromatin were largely unaffected by lack of EZH2 activity (Figure 5A). The definition of H3K27me3 domain borders and pattern of H3K27me3 domains were unchanged in the presence of inhibitor, indicating that H3K27me3 positional information was maintained post replication (Figures 5B and 5C). These results demonstrate that the nascent H3K27me3 landscape is the result of parental histone recycling, with little, if any, contribution from *de novo* methylation events.

We next compared our T0 and T24 data, both with and without inhibitor. Signal comparison between all samples revealed that the accumulation of H3K27me3 during chromatin maturation was entirely dependent on *de novo* H3K27 methylation (Figure 5D), as predicted. Unexpectedly, however, the H3K27me3 landscape generated from parental histone recycling at the time of replication persisted in daughter cells 24 hr post DNA

replication (Figures 5E and 5F), arguing that nucleosome turnover and/or demethylation do not generally erode the H3K27me3 landscape.

To investigate whether sustained EZH2 inhibition causes H3K27me3 loss in certain local genomic regions, we defined regions of low, moderate, and high H3K27me3 loss by comparing qChOR-seq signal at T0 and T24 from EZH2 inhibitor-treated cells (see STAR Methods). While the large majority of H3K27me3 loci did not change substantially, 3.5% of the regions showed a moderate reduction in H3K27me3 (Figure 5G). These regions of moderate H3K27me3 loss were characterized by somewhat lower H3K27me3 and EZH2 occupancy compared to the more stable regions (Figure 5H). In *Drosophila*, H3K27me3-marked regions, such as Polycomb group response elements, have been identified as fragile high-turnover sites (Deal et al., 2010). However, in our inhibitor-treated HeLa cells, regions of moderate H3K27me3 loss did not correlate with higher occupancy of the replacement histone variant H3.3 (Figure S5A), suggesting that nucleosome turnover is not sufficient to explain the decrease in signal observed. Taken together, H3K27me3 qChOR-seq in the absence of *de novo* H3K27 methylation revealed that domains laid down at the time of replication are remarkably stable, underscoring histone recycling during DNA replication as a substantial contributor to the inheritance of H3K27me3 in daughter cells without challenges by demethylation or histone exchange.

DISCUSSION

Here we develop ChOR-seq to determine the occupancy of modified histones on replicated DNA. We track methylated histones associated with active and repressed chromatin and find that their position on newly replicated DNA mirrors their position prior to replication. This demonstrates that the histone modification landscape can withstand the disruptive process of DNA replication. Several lines of evidence point toward accurate recycling of modified parental histones as the underlying mechanism. First, new histones are devoid of tri-methylation (Alabert et al., 2015; Bar-Ziv et al., 2016; Scharf et al., 2009; Xu et al., 2011), arguing that we mainly detect pre-existing marks on old recycled histones. Second, H3K27me3 occupancy patterns are accurately copied in the absence of EZH2 activity. Third, quantitative ChOR-seq analysis showed a strong increase in H3K4me3 and H3K27me3 signal intensity during chromatin maturation, arguing that *de novo* tri-methylation occurs in a manner uncoupled from DNA replication. We did not detect dispersal of either H3K4me3, H3K36me3, H3K79me3, or H3K27me3 marks as a result of DNA replication, which would be the predicted outcome if parental histones were either fully released and re-incorporated at a different replication fork or maintained loosely at the fork and re-deposited haphazardly. Of note, canonical histones H3.1/2 and the replacement variant H3.3 are not differentiated in our analysis because they are recycled with equal efficiency during DNA replication and deposited unmodified *de novo* (Alabert et al., 2015; Jasencakova et al., 2010; Loyola et al., 2006). Thus, all histone H3 variants can contribute to pre-marked parental histones in nascent chromatin and be subject to *de novo* methylation during chromatin restoration. A recent *in vitro* study found that histone positioning information is lost in *Xenopus* extracts when large T antigen acts as the replicative helicase (Madamba et al., 2017) and suggested that dispersal of histones might be more limited when replication is mediated by the MCM2-7 helicase, as it is in human cells. Our data show that parental histones are re-incorporated within 250 bp of their original position in human cells, which argues that histone eviction and re-deposition at the fork must be tightly coordinated. In human cells, histone-based information is thus retained with higher precision than predicted by mathematic modeling in yeast (Radman-Livaja et al., 2011) and observed in *Xenopus in vitro* replication systems (Madamba et al., 2017), two systems in which replication-independent histone exchange is high. Collectively, this argues that recycling of parental histones at the replication fork is highly accurate, reproducing the landscape of histone modifications on newly synthesized DNA, albeit with a lower amplitude due to dilution by new naive histones.

Combining a spike-in approach with ChOR-seq, we were able to measure with base-pair resolution how histone mark levels recovered after DNA replication. The strength of spike-in ChOR-seq is that it allows quantitative comparison of restoration kinetics across the genome. Using ChOR-seq to obtain spatial information, we found that the site of occupancy is fixed at the time of replication and modifications then accumulate with kinetics inherent to the mark and genomic features of the locus as cells progress through the cell cycle. Mass spectrometry analysis had previously identified H3K27me3 and H3K9me3 as particularly slow-recovering marks post replication (Alabert et al., 2015; Scharf et al., 2009; Xu et al., 2011). ChOR-seq corroborated these results and further revealed that restoration kinetics are not uniform throughout the genome. While restoration of most H3K27me3 domains is slow, continuing after mitosis in daughter cells, sites of high H3K27me3 and PRC2 occupancy recover faster than other regions. Likewise, our results indicate that sites with the highest H3K4me3 levels, corresponding to CpG-dense, highly expressed promoters (Chen et al., 2014; Illingworth et al., 2010; Mikkelsen et al., 2007), also are first to gain H3K4me3 after replication. Our results show that the vast majority of H3K4me3 peaks are restored prior to mitosis. This means that the H3K4me3 landscape transmitted to daughter cells is not affected by differential restoration rates, in contrast to the H3K27me3 landscape where the sites of the highest H3K27me3 and PRC2 occupancy have gained relatively more signal than other regions. This may not be determined solely by methyltransferase kinetics but could also be influenced by replication timing, since early-replicating chromatin is gene rich and transcriptionally active while late-replicating chromatin tends to be heterochromatic (Rivera-Mulia and Gilbert, 2016). Importantly, it argues that the epigenome is not fixed for a given cell but should be considered as a dynamic landscape changing throughout the cell cycle with regards to the total level of all marks, the relative enrichment of individual marks across different sites, and the abundance of different marks relative to each other.

Even though the correct position and relative abundance of histone marks are maintained during DNA replication, new histone deposition represents a major challenge to the epigenome,

with histone-modifying enzymes required to counteract replication-induced erosion of the landscape. Because many of these enzymes, such as EZH2, act with considerable delay, this leads to heterogeneity in the histone-modification landscape across the cell cycle. We suggest that this should be investigated as a possible source of cell-to-cell heterogeneity in gene expression, differentiation potential, and diseases such as cancer. Because the abundance, activity, and complex composition of histone-modifying enzymes, as well as cell cycle duration, varies across cell types, restoration kinetics are expected to be cell-type specific. Importantly, ChOR-seq can easily be applied to any protein or modification amenable to ChIP, thus providing a means to address replication-dependent epigenome heterogeneity and how it impacts cell-fate decisions.

Several elegant studies in model organisms have shown that histone modifications can be transmitted to daughter cells in the absence of the modifying enzyme, although the marks are progressively diluted over time (Audergon et al., 2015; Coleman and Struhl, 2017; Gaydos et al., 2014; Laprell et al., 2017; Ragunathan et al., 2015). Our findings reveal that histone-occupancy patterns are accurately copied during DNA replication prior to their transmission to daughter cells in mitosis. This is consistent with recent findings that impaired DNA replication can have major epigenetic consequences, changing gene expression and generating epi-alleles (Klosin et al., 2017; Sarkies et al., 2010). However, two studies have suggested that histone modifications are erased at the time of DNA replication and must be established *de novo* on new and old histones after DNA replication (Petruk et al., 2012, 2013). The investigators relied on a proximity-ligation assay to indicate the presence of modified histones on replicated DNA in *Drosophila* embryos. Our results, along with evidence from genetic model systems (Audergon et al., 2015; Coleman and Struhl, 2017; Gaydos et al., 2014; Hansen et al., 2008; Laprell et al., 2017; Ragunathan et al., 2015) and mass spectrometry analysis of marks on new and old histones (Alabert et al., 2015; Pesavento et al., 2008; Scharf et al., 2009; Xu et al., 2011; Zee et al., 2012), contradict with this conclusion. We suspect that this mainly reflects the lack of sensitivity of the proximity-based ligation assay, underscoring ChOR-seq as a superior technology to track modification on replicated DNA and provide highly sensitive site-specific and quantitative information. In fact, while our work was under consideration, a technology similar to ChOR-seq to track proteins on newly replicated DNA was published by Xu and Corces (nasChIP-seq) (Xu and Corces, 2018a, 2018b). NasChIP-seq and ChOR-seq have the potential to rapidly advance our understanding of the post-replicative chromatin environment and epigenome maintenance in general.

Modified old histones re-instated at their original position after DNA replication could contribute to epigenetic cell memory in two ways: by preserving the properties of the parental chromatin state and by positive feedforward stimulation of modification on new neighboring histones to facilitate restoration. Therefore, our demonstration that old modified histones are re-instated at their original position after DNA replication provides a significant advance in understanding how histone marks could contribute to epigenetic cell memory. Notably, we find that parental H3K27me3 domains not only withstand DNA replication, but they remain stable across the cell cycle, underscoring that recycled parental histones shape the chromatin environment in daughter cells. Future landmarks will include an understanding of whether histones H2A-H2B follow a similar strict pattern of transmission during DNA replication and whether modified histones are distributed symmetrically on the two daughter stands.

STAR★METHODS

Detailed methods are provided in the online version of this paper and include the following:

- KEY RESOURCES TABLE
- CONTACT FOR REAGENT AND RESOURCE SHARING
- EXPERIMENTAL MODEL AND SUBJECT DETAILS
- METHOD DETAILS
  - Synchronization and DNA Labeling
  - ChOR-Seq, ChIP-Seq, and Replicated DNA Isolation
  - Data Sequencing and Processing
  - PTM Distribution Analyses
  - Restoration Categories and Analyses
  - Cell Cycle Analyses
- QUANTIFICATION AND STATISTICAL ANALYSIS
- DATA AND SOFTWARE AVAILABILITY

SUPPLEMENTAL INFORMATION

Supplemental Information includes five figures and can be found with this article online at https://doi.org/10.1016/j.molcel.2018.08.010.

ACKNOWLEDGMENTS

We would like to thank the K. Helin laboratory for sharing reagents and B. Leblanc and the Groth laboratory for fruitful discussion. We also thank the ENCODE Consortium and the Stamatoyannopoulous' laboratory, University of Washington, for the Repli-seq data. This work was supported by post doc fellowships from the Lundbeck Foundation to C.G.-A., N.R.-G. (R165-2013-15306), and K.R.S.-M. (R219-2016-394). Research in the Groth lab was supported by the Lundbeck Foundation (R198-2015-269), the Independent Research Fund Denmark (7016-00042B and 4092-00404B), the European Research Council (ERC CoG no. 724436), the Danish National Research Foundation to the Center for Epigenetics (DNRF82), the Danish Cancer Society, the Novo Nordisk Foundation, and the NEYE Foundation.

AUTHOR CONTRIBUTIONS

N.R.-G., C.G.-A., K.R.S.-M., and A.G. conceived the project and designed experiments. C.A. helped to conceive the project. N.R.-G. and C.G.-A. performed the experiments and data analysis. N.R.-G., C.G.-A., and N.P. developed the ChOR-seq protocol. V.F. and K.R.S.-M. contributed to ChOR-seq experiments for active marks. K.R.S.-M. contributed to data analysis and discussions and performed CG content analysis for H3K4me3 experiments. S.G. helped with control experiments for EZH2i. J.V.J. and J.S.J. helped with data analysis and protocol development, respectively. K.R.S.-M., C.G.-A., and A.G. wrote the manuscript with inputs from all authors.

DECLARATION OF INTERESTS

The authors declare no competing interests.

Received: March 20, 2018
Revised: June 25, 2018
Accepted: August 7, 2018
Published: August 23, 2018

REFERENCES

Afgan, E., Baker, D., van den Beek, M., Blankenberg, D., Bouvier, D., Čech, M., Chilton, J., Clements, D., Coraor, N., Eberhard, C., et al. (2016). The Galaxy platform for accessible, reproducible and collaborative biomedical analyses: 2016 update. Nucleic Acids Res. *44* (W1), W3–W10.

Alabert, C., and Groth, A. (2012). Chromatin replication and epigenome maintenance. Nat. Rev. Mol. Cell Biol. *13*, 153–167.

Alabert, C., Bukowski-Wills, J.C., Lee, S.B., Kustatscher, G., Nakamura, K., de Lima Alves, F., Menard, P., Mejlvang, J., Rappsilber, J., and Groth, A. (2014). Nascent chromatin capture proteomics determines chromatin dynamics during DNA replication and identifies unknown fork components. Nat. Cell Biol. *16*, 281–293.

Alabert, C., Barth, T.K., Reverón-Gómez, N., Sidoli, S., Schmidt, A., Jensen, O.N., Imhof, A., and Groth, A. (2015). Two distinct modes for propagation of histone PTMs across the cell cycle. Genes Dev. *29*, 585–590.

Allis, C.D., and Jenuwein, T. (2016). The molecular hallmarks of epigenetic control. Nat. Rev. Genet. *17*, 487–500.

Almouzni, G., and Cedar, H. (2016). Maintenance of epigenetic information. Cold Spring Harb. Perspect. Biol. *8*, 8.

Anders, S. (2009). Visualization of genomic data with the Hilbert curve. Bioinformatics *25*, 1231–1235.

Annunziato, A.T. (2015). The fork in the road: histone partitioning during DNA replication. Genes (Basel) *6*, 353–371.

Audergon, P.N., Catania, S., Kagansky, A., Tong, P., Shukla, M., Pidoux, A.L., and Allshire, R.C. (2015). Epigenetics. Restricted epigenetic inheritance of H3K9 methylation. Science *348*, 132–135.

Bar-Ziv, R., Voichek, Y., and Barkai, N. (2016). Chromatin dynamics during DNA replication. Genome Res. *26*, 1245–1256.

Bernstein, B.E., Kamal, M., Lindblad-Toh, K., Bekiranov, S., Bailey, D.K., Huebert, D.J., McMahon, S., Karlsson, E.K., Kulbokas, E.J., 3rd, Gingeras, T.R., et al. (2005). Genomic maps and comparative analysis of histone modifications in human and mouse. Cell *120*, 169–181.

Bonhoure, N., Bounova, G., Bernasconi, D., Praz, V., Lammers, F., Canella, D., Willis, I.M., Herr, W., Hernandez, N., and Delorenzi, M.; CycliX Consortium (2014). Quantifying ChIP-seq data: a spiking method providing an internal reference for sample-to-sample normalization. Genome Res. *24*, 1157–1168.

Brown, D.A., Di Cerbo, V., Feldmann, A., Ahn, J., Ito, S., Blackledge, N.P., Nakayama, M., McClellan, M., Dimitrova, E., Turberfield, A.H., et al. (2017). The SET1 complex selects actively transcribed target genes via multivalent interaction with CpG island chromatin. Cell Rep. *20*, 2313–2327.

Campos, E.I., Stafford, J.M., and Reinberg, D. (2014). Epigenetic inheritance: histone bookmarks across generations. Trends Cell Biol. *24*, 664–674.

Chen, R.A., Stempor, P., Down, T.A., Zeiser, E., Feuer, S.K., and Ahringer, J. (2014). Extreme HOT regions are CpG-dense promoters in C. elegans and humans. Genome Res. *24*, 1138–1146.

Coleman, R.T., and Struhl, G. (2017). Causal role for inheritance of H3K27me3 in maintaining the OFF state of a *Drosophila* HOX gene. Science *356*, 356.

Comet, I., Riising, E.M., Leblanc, B., and Helin, K. (2016). Maintaining cell identity: PRC2-mediated regulation of transcription and cancer. Nat. Rev. Cancer *16*, 803–810.

Comoglio, F., and Paro, R. (2014). Combinatorial modeling of chromatin features quantitatively predicts DNA replication timing in Drosophila. PLoS Comput. Biol. *10*, e1003419.

Deal, R.B., Henikoff, J.G., and Henikoff, S. (2010). Genome-wide kinetics of nucleosome turnover determined by metabolic labeling of histones. Science *328*, 1161–1164.

ENCODE Project Consortium (2012). An integrated encyclopedia of DNA elements in the human genome. Nature *489*, 57–74.

Fennessy, R.T., and Owen-Hughes, T. (2016). Establishment of a promoter-based chromatin architecture on recently replicated DNA can accommodate variable inter-nucleosome spacing. Nucleic Acids Res. *44*, 7189–7203.

Gasser, R., Koller, T., and Sogo, J.M. (1996). The stability of nucleosomes at the replication fork. J. Mol. Biol. *258*, 224–239.

Gaydos, L.J., Wang, W., and Strome, S. (2014). Gene repression. H3K27me and PRC2 transmit a memory of repression across generations and during development. Science *345*, 1515–1518.

Gruss, C., Wu, J., Koller, T., and Sogo, J.M. (1993). Disruption of the nucleosomes at the replication fork. EMBO J. *12*, 4533–4545.

Halley-Stott, R.P., and Gurdon, J.B. (2013). Epigenetic memory in the context of nuclear reprogramming and cancer. Brief. Funct. Genomics *12*, 164–173.

Hansen, K.H., Bracken, A.P., Pasini, D., Dietrich, N., Gehani, S.S., Monrad, A., Rappsilber, J., Lerdrup, M., and Helin, K. (2008). A model for transmission of the H3K27me3 epigenetic mark. Nat. Cell Biol. *10*, 1291–1300.

Højfeldt, J.W., Laugesen, A., Willumsen, B.M., Damhofer, H., Hedehus, L., Tvardovskiy, A., Mohammad, F., Jensen, O.N., and Helin, K. (2018). Accurate H3K27 methylation can be established *de novo* by SUZ12-directed PRC2. Nat. Struct. Mol. Biol. *25*, 225–232.

Huber, W., Carey, V.J., Gentleman, R., Anders, S., Carlson, M., Carvalho, B.S., Bravo, H.C., Davis, S., Gatto, L., Girke, T., et al. (2015). Orchestrating high-throughput genomic analysis with Bioconductor. Nat. Methods *12*, 115–121.

Illingworth, R.S., Gruenewald-Schneider, U., Webb, S., Kerr, A.R., James, K.D., Turner, D.J., Smith, C., Harrison, D.J., Andrews, R., and Bird, A.P. (2010). Orphan CpG islands identify numerous conserved promoters in the mammalian genome. PLoS Genet. *6*, e1001134.

Jackson, V. (1987). Deposition of newly synthesized histones: new histones H2A and H2B do not deposit in the same nucleosome with new histones H3 and H4. Biochemistry *26*, 2315–2325.

Jackson, V. (1988). Deposition of newly synthesized histones: hybrid nucleosomes are not tandemly arranged on daughter DNA strands. Biochemistry *27*, 2109–2120.

Jackson, V. (1990). In vivo studies on the dynamics of histone-DNA interaction: evidence for nucleosome dissolution during replication and transcription and a low level of dissolution independent of both. Biochemistry *29*, 719–731.

Jakobsen, J.S., Waage, J., Rapin, N., Bisgaard, H.C., Larsen, F.S., and Porse, B.T. (2013). Temporal mapping of CEBPA and CEBPB binding during liver regeneration reveals dynamic occupancy and specific regulatory codes for homeostatic and cell cycle gene batteries. Genome Res. *23*, 592–603.

Jasencakova, Z., Scharf, A.N.D., Ask, K., Corpet, A., Imhof, A., Almouzni, G., and Groth, A. (2010). Replication stress interferes with histone recycling and predeposition marking of new histones. Mol. Cell *37*, 736–743.

Jiao, L., and Liu, X. (2015). Structural basis of histone H3K27 trimethylation by an active polycomb repressive complex 2. Science *350*, aac4383.

Julienne, H., Zoufir, A., Audit, B., and Arneodo, A. (2013). Human genome replication proceeds through four chromatin states. PLoS Comput. Biol. *9*, e1003233.

Kim, J., Cantor, A.B., Orkin, S.H., and Wang, J. (2009). Use of in vivo biotinylation to study protein-protein and protein-DNA interactions in mouse embryonic stem cells. Nat. Protoc. *4*, 506–517.

Klosin, A., Reis, K., Hidalgo-Carcedo, C., Casas, E., Vavouri, T., and Lehner, B. (2017). Impaired DNA replication derepresses chromatin and generates a transgenerationally inherited epigenetic memory. Sci. Adv. *3*, e1701143.

Knutson, S.K., Warholic, N.M., Wigle, T.J., Klaus, C.R., Allain, C.J., Raimondi, A., Porter Scott, M., Chesworth, R., Moyer, M.P., Copeland, R.A., et al. (2013). Durable tumor regression in genetically altered malignant rhabdoid tumors by inhibition of methyltransferase EZH2. Proc. Natl. Acad. Sci. USA *110*, 7922–7927.

Kulyyassov, A., Shoaib, M., and Ogryzko, V. (2011). Use of in vivo biotinylation for chromatin immunoprecipitation. Curr. Protoc. Cell Biol. *51*, 17.12.1–17.12.22.

Langmead, B., Trapnell, C., Pop, M., and Salzberg, S.L. (2009). Ultrafast and memory-efficient alignment of short DNA sequences to the human genome. Genome Biol. *10*, R25.

Laprell, F., Finkl, K., and Müller, J. (2017). Propagation of Polycomb-repressed chromatin requires sequence-specific recruitment to DNA. Science *356*, 85–88.

Loyola, A., Bonaldi, T., Roche, D., Imhof, A., and Almouzni, G. (2006). PTMs on H3 variants before chromatin assembly potentiate their final epigenetic state. Mol. Cell *24*, 309–316.

Madamba, E.V., Berthet, E.B., and Francis, N.J. (2017). Inheritance of histones H3 and H4 during DNA replication in vitro. Cell Rep. *21*, 1361–1374.

Margueron, R., Justin, N., Ohno, K., Sharpe, M.L., Son, J., Drury, W.J., 3rd, Voigt, P., Martin, S.R., Taylor, W.R., De Marco, V., et al. (2009). Role of the polycomb protein EED in the propagation of repressive histone marks. Nature *461*, 762–767.

McKnight, S.L., and Miller, O.L., Jr. (1977). Electron microscopic analysis of chromatin replication in the cellular blastoderm Drosophila melanogaster embryo. Cell *12*, 795–804.

Mikkelsen, T.S., Ku, M., Jaffe, D.B., Issac, B., Lieberman, E., Giannoukos, G., Alvarez, P., Brockman, W., Kim, T.K., Koche, R.P., et al. (2007). Genome-wide maps of chromatin state in pluripotent and lineage-committed cells. Nature *448*, 553–560.

Mortazavi, A., Williams, B.A., McCue, K., Schaeffer, L., and Wold, B. (2008). Mapping and quantifying mammalian transcriptomes by RNA-seq. Nat. Methods *5*, 621–628.

Orlando, D.A., Chen, M.W., Brown, V.E., Solanki, S., Choi, Y.J., Olson, E.R., Fritz, C.C., Bradner, J.E., and Guenther, M.G. (2014). Quantitative ChIP-seq normalization reveals global modulation of the epigenome. Cell Rep. *9*, 1163–1170.

Patel, D.J., and Wang, Z. (2013). Readout of epigenetic modifications. Annu. Rev. Biochem. *82*, 81–118.

Pesavento, J.J., Yang, H., Kelleher, N.L., and Mizzen, C.A. (2008). Certain and progressive methylation of histone H4 at lysine 20 during the cell cycle. Mol. Cell. Biol. *28*, 468–486.

Petruk, S., Sedkov, Y., Johnston, D.M., Hodgson, J.W., Black, K.L., Kovermann, S.K., Beck, S., Canaani, E., Brock, H.W., and Mazo, A. (2012). TrxG and PcG proteins but not methylated histones remain associated with DNA through replication. Cell *150*, 922–933.

Petruk, S., Black, K.L., Kovermann, S.K., Brock, H.W., and Mazo, A. (2013). Stepwise histone modifications are mediated by multiple enzymes that rapidly associate with nascent DNA during replication. Nat. Commun. *4*, 2841.

Pope, B.D., Ryba, T., Dileep, V., Yue, F., Wu, W., Denas, O., Vera, D.L., Wang, Y., Hansen, R.S., Canfield, T.K., et al. (2014). Topologically associating domains are stable units of replication-timing regulation. Nature *515*, 402–405.

Presolski, S.I., Hong, V.P., and Finn, M.G. (2011). Copper-catalyzed azide-alkyne click chemistry for bioconjugation. Curr. Protoc. Chem. Biol. *3*, 153–162.

Radman-Livaja, M., Verzijlbergen, K.F., Weiner, A., van Welsem, T., Friedman, N., Rando, O.J., and van Leeuwen, F. (2011). Patterns and mechanisms of ancestral histone protein inheritance in budding yeast. PLoS Biol. *9*, e1001075.

Ragunathan, K., Jih, G., and Moazed, D. (2015). Epigenetics. Epigenetic inheritance uncoupled from sequence-specific recruitment. Science *348*, 1258699.

Ramachandran, S., and Henikoff, S. (2016). Transcriptional regulators compete with nucleosomes post-replication. Cell *165*, 580–592.

Rando, O.J. (2007). Global patterns of histone modifications. Curr. Opin. Genet. Dev. *17*, 94–99.

Ray-Gallet, D., Woolfe, A., Vassias, I., Pellentz, C., Lacoste, N., Puri, A., Schultz, D.C., Pchelintsev, N.A., Adams, P.D., Jansen, L.E.T., and Almouzni, G. (2011). Dynamics of histone H3 deposition in vivo reveal a nucleosome gap-filling mechanism for H3.3 to maintain chromatin integrity. Mol. Cell *44*, 928–941.

Rivera-Mulia, J.C., and Gilbert, D.M. (2016). Replication timing and transcriptional control: beyond cause and effect-part III. Curr. Opin. Cell Biol. *40*, 168–178.

Sarkies, P., Reams, C., Simpson, L.J., and Sale, J.E. (2010). Epigenetic instability due to defective replication of structured DNA. Mol. Cell *40*, 703–713.

Scharf, A.N., Meier, K., Seitz, V., Kremmer, E., Brehm, A., and Imhof, A. (2009). Monomethylation of lysine 20 on histone H4 facilitates chromatin maturation. Mol. Cell. Biol. *29*, 57–67.

Schuettengruber, B., Bourbon, H.M., Di Croce, L., and Cavalli, G. (2017). Genome regulation by Polycomb and Trithorax: 70 years and counting. Cell *171*, 34–57.

Stempor, P., and Ahringer, J. (2016). SeqPlots - interactive software for exploratory data analyses, pattern discovery and visualization in genomics. Wellcome Open Res. https://doi.org/10.12688/wellcomeopenres.10004.1.

Vasseur, P., Tonazzini, S., Ziane, R., Camasses, A., Rando, O.J., and Radman-Livaja, M. (2016). Dynamics of nucleosome positioning maturation following genomic replication. Cell Rep. *16*, 2651–2665.

Xu, C., and Corces, V.G. (2018a). Genome-Wide Mapping of Protein-DNA Interactions on Nascent Chromatin. Methods Mol. Biol. *1766*, 231–238.

Xu, C., and Corces, V.G. (2018b). Nascent DNA methylome mapping reveals inheritance of hemimethylation at CTCF/cohesin sites. Science *359*, 1166–1170.

Xu, M., Long, C., Chen, X., Huang, C., Chen, S., and Zhu, B. (2010). Partitioning of histone H3-H4 tetramers during DNA replication-dependent chromatin assembly. Science *328*, 94–98.

Xu, M., Wang, W., Chen, S., and Zhu, B. (2011). A model for mitotic inheritance of histone lysine methylation. EMBO Rep. *13*, 60–67.

Zee, B.M., Britton, L.M., Wolle, D., Haberman, D.M., and Garcia, B.A. (2012). Origins and formation of histone methylation across the human cell cycle. Mol. Cell. Biol. *32*, 2503–2514.

Zhang, Y., Liu, T., Meyer, C.A., Eeckhoute, J., Johnson, D.S., Bernstein, B.E., Nusbaum, C., Myers, R.M., Brown, M., Li, W., and Liu, X.S. (2008). Model-based analysis of ChIP-seq (MACS). Genome Biol. *9*, R137.

STAR★METHODS

KEY RESOURCES TABLE

REAGENT or RESOURCE	SOURCE	IDENTIFIER
Antibodies		
H3K27me3 C36B11	Cell Signaling Technology	Cat No. 9733S; RRID: AB_2616029
Total H3	Abcam	Cat No. Ab1791; RRID: AB_302613
H3K4me3 C42D8	Cell Signaling Technology	Cat No. 9751S; RRID: AB_2616028
Histone H3 (tri methyl K36) antibody - ChIP Grade	Abcam	Cat No. ab9050; RRID: AB_306966
Anti-Histone H3 (tri methyl K79) antibody - ChIP Grade, purified	Abcam	Cat No. ab195500
Chemicals, Peptides, and Recombinant Proteins		
DMEM	Thermo Fisher Scientific	Cat No. 11965092
FBS	Invitrogen	Cat No. 26400-036
Shields and Sang M3 insect medium	Sigma-Aldrich	Cat No. S8398
EdU	Invitrogen	Cat No. A10044
Biotin-16-dUTP	IBA oligonucleotides	N/A
Biotin-TEG-Azide	Berry & Associates	Cat No. BT1085
Free Biotin	Sigma-Aldrich	Cat No. B4501
THPTA	Sigma-Aldrich	Cat No. 762342
Aminoguanidine hydrochloride	Sigma-Aldrich	Cat No. 396494
Protein A agarose beads	Thermo Fisher Scientific	Cat No. 20333
Dynabeads MyOne Streptavidin T1	Thermo Fisher Scientific	Cat No. 65602
EPZ-6438 EZH2 inhibitor	Selleckchem	Cat No.S7128
Agencourt AMPure XP	Beckman Coulter	Cat No. A63880
Critical Commercial Assays		
Click-IT Alexa Fluor 488 imaging kit	Thermo Fisher Scientific	Cat No. C10337
QIAGEN MiniElute PCR purification kit	QIAGEN	Cat No. 28004
NEB Next Ultra DNA Library prep kit	New England Biolabs	Cat No. NEB E7370S
KAPA Hyperprep Kit	Kappa Biosystems, Roche	Cat No. KK8504
Deposited Data		
Repli-seq	ENCODE Project Consortium, 2012	GEO: GSM923449
Encode HeLa S3 H3K27me3 ChIP	Bernstein et al., 2005	GEO: GSM733696
Encode HeLa S3 H3K4me3 ChIP	Bernstein et al., 2005	GEO: GSM733682
HeLa S3 EZH2 ChIP	Bernstein et al., 2005	GEO: GSM1003520
HeLa S3 RNaseq	Mortazavi et al., 2008	GEO: GSM958735
HeLa H3.3 ChIP	Ray-Gallet et al., 2011	GEO: GSM788633
TSSs	http://hgdownload.soe.ucsc.edu/goldenPath/hg19/encodeDCC/wgEncodeCaltechRnaSeq/	wgEncodeCaltechRnaSeqHelas3R2x75Il200TSSRep1V3.gtf.gz
RefSeq	http://hgdownload.soe.ucsc.edu/goldenPath/hg19/database/	refGene.txt.gz
CpG densities	Illingworth et al., 2010	https://doi.org/10.1371/journal.pgen.1001134.s001
Raw and analyzed data	This paper	GEO: GSE110354

(*Continued on next page*)

Continued		
REAGENT or RESOURCE	SOURCE	IDENTIFIER
Experimental Models: Cell lines		
Human HeLa S3 cells	ATCC	Cat. No. CCL-2-2; RRID: CVCL_0058
Drosophila S2-DRSC cells	Drosophila Genomics Resource Center; Stock No. 181	RRID: CVCL_Z992
Oligonucleotides		
NGS indexed PentAdapters	PentaBase	Cat No. SKU 310
Software and Algorithms		
Bowtie 1	Langmead et al., 2009	http://bowtie-bio.sourceforge.net/index.shtml
Galaxy	Afgan et al., 2016	RRID: SCR_006281; https://galaxyproject.org/
MACS	Zhang et al., 2008	http://liulab.dfci.harvard.edu/MACS/
R v.3.2.1	Huber et al., 2015	https://www.bioconductor.org/
SeqPlots v.1	Stempor and Ahringer, 2016	https://bioconductor.org/packages/release/bioc/html/seqplots.html
Hilbertvis v.3.6	Anders, 2009	RRID: SCR_007862; https://bioconductor.org/packages/release/bioc/html/HilbertVis.html

CONTACT FOR REAGENT AND RESOURCE SHARING

Further information and request for resources and reagents should be directed to and will be fulfilled by the Lead Contact Anja Groth (anja.groth@bric.ku.dk).

EXPERIMENTAL MODEL AND SUBJECT DETAILS

Human HeLa S3 cells (female) were obtained from ATCC and were grown in suspension in spinners. Cells were grown in DMEM medium (Thermo Fisher Scientific) supplemented with dialyzed FBS (Invitrogen), penicillin, and streptomycin at 37°C and 5% CO_2. *Drosophila* S2-DRSC cells were obtained from the *Drosophila* Genomics Resource Center. S2 cells were grown in suspension in spinners in M3+BPYE (Sigma-Aldrich) media with 10% heat-inactivated FBS, penicillin and streptomycin at 25°C.

METHOD DETAILS

Synchronization and DNA Labeling

For all ChOR-seq and ChIP-seq, HeLa S3 cells were synchronized at the G1/S border by a single thymidine block (2 mM, 17 hr) and released into fresh media containing deoxycytidine (24 μM) for the indicated amount of time. For H3K4me3, H3K36me3 and H3K79me3 analysis, parental samples were collected immediately after release. 2 hr and 15 min later (early S), DNA was labeled for 10 min with medium containing 5-ethynyl-2′-deoxyuridine (EdU, 10 μM). Immediately following the EdU label, nascent chromatin was collected. To study H3K4me3 maturation, following EdU labeling and collection of a subset of cells as the “nascent” sample, remaining cells in the spinner culture were washed with PBS, resuspended in new medium containing deoxycytidine (24 μM), and incubated at 37°C until collection at the indicated time point.

For H3K27me3 analysis, the same procedure as above was followed, but with different time points. The parental sample was collected 2 hr and 15 min after release from the thymidine block. 1 hr later (mid S), DNA was labeled for 20 min with medium containing EdU (10 μM). Immediately following the EdU label, nascent chromatin was collected. To study H3K27me3 maturation, following EdU labeling and collection of a subset of cells as the “nascent” sample, remaining cells in the spinner culture were washed with PBS, resuspended in new medium containing deoxycytidine (24 μM), and incubated at 37°C until collection at the indicated time point. To prevent cells from entering into a second round of replication, thymidine (2mM) was added to the medium 10 hr post-EdU label and cells were incubated until reaching 24 hr post-EdU label. For experiments using EZH2 inhibitor (EPZ-6438, 1 μM) (Knutson et al., 2013), inhibitor was added to the medium 30 min before collecting nascent chromatin and kept in culture medium until the end of the experiment.

For qChOR-seq experiments, asynchronous *Drosophila* S2 chromatin was labeled with EdU (10 μM) for 39 hr for use as an internal control. For Figures S1G and S1H, ChOR-seq was carried out without exogenous *Drosophila* chromatin and newly synthesized DNA was labeled with biotin-dUTP as described in Alabert et al. (2014). Briefly, cells were labeled with biotin-dUTP for 5 min in hypotonic buffer (50 mM KCl, 10 mM HEPES) followed by a further 15 min in fresh media with biotin-dUTP and deoxycytidine.

ChOR-Seq, ChIP-Seq, and Replicated DNA Isolation

In ChOR-seq, ChIP-seq and replicated DNA isolation, HeLa S3 cells were immediately fixed in 1% formaldehyde. Then, glycine was added to a final concentration of 0.125 M and the reaction was incubated for 5 min at room temperature. Fixed cells were lysed for 20 min in ice-cold lysis buffer (100 mM NaCl, 66 mM Tris-HCl pH 8, 5 mM EDTA, 0.3% SDS, 1.6% Triton X-100) supplemented with leupeptin, aprotinin, pepstatin, and PMSF. Lysates were passed through a 21G needle and sonicated using a Bioruptor nextGen (Diagenode) with the following settings: 20 cycles, 30 s ON, 30 s OFF, high intensity. Sonicated chromatin was centrifuged at 14,000 rpm at 4°C for 10 min and the supernatant was isolated for subsequent steps. In parallel, *Drosophila* S2 cells were fixed, lysed, and sonicated as described above. After sonication, HeLa S3 input chromatin was mixed with *Drosophila* S2 chromatin (0.025 to 2.5% of total chromatin).

Parental ChIPs were performed as described in Jakobsen et al. (2013). In brief, 30 μg total of mixed HeLa S3 and *Drosophila* S2 sonicated chromatin were diluted up to 500 μL with dialysis buffer (4% glycerol, 10 mM Tris-HCl, 1 mM EDTA, 0.5 mM EGTA; pH 8) and 400 μL of incubation buffer (2.5% Triton X-100, 0.25% sodium deoxycholate, 0.25% SDS, 0.35 M NaCl, 10 mM Tris-HCl; pH 8) supplemented with leupeptin, aprotinin, pepstatin, and PMSF. Chromatin was pre-cleared with Protein A agarose beads for 1 hr at 4°C. After pre-clearing, chromatin was incubated with the corresponding antibody (H3K4me3: C42D8, Cell Signaling Technology; H3K36me3: ab9050, Abcam; H3K79me3: ab195500, Abcam; H3K27me3: C36B11, Cell Signaling Technology; H3: ab1791, Abcam) overnight at 4°C, followed by incubation for 3 hr with pre-blocked Protein A agarose beads (incubated in 1 mg/ml BSA in RIPA buffer overnight). Chromatin bound to beads was washed three times in RIPA buffer (140 mM NaCl, 10 mM Tris-HCl, 1 mM EDTA, 1% Triton X-100, 0.1% SDS, 0.1% sodium deoxycholate, 1 mM PMSF; pH 8), once in RIPA buffer with 0.5 M NaCl, once in LiCl buffer (250 mM LiCl, 10 mM Tris-HCl, 1 mM EDTA, 0.5% NP-40, 0.5% sodium deoxycholate; pH 8) and twice in TE (10 mM Tris-HCl, 1 mM EDTA; pH 8). Chromatin was incubated with RNase A for 30 min at 37°C. SDS was then added to a final concentration of 0.5% and samples were incubated with proteinase K (1 mg/ml) for 10 hr at 37°C followed by 6 hr incubation at 65°C for de-crosslinking. DNA was purified using the MinElute PCR purification kit (QIAGEN). Finally, 10 ng of purified DNA was subjected to end repair, A-tailing and amplification using the KAPA Hyperprep kit protocol (Roche). Before amplification, DNA was size-selected with Agencourt AMPure XP beads (Beckman Coulter) to obtain fragments between 200-700 bp. For amplification, 6 PCR cycles were used followed by clean-up with Agencourt AMPure XP beads.

For ChOR-seq experiments, 30 ug total of sonicated HeLa S3 and *Drosophila* S2 mixed chromatin was subjected to standard ChIP as described above. 100 ng of immunoprecipitated DNA or de-crosslinked input material from labeled chromatin was then subjected to end repair, A-tailing, and adaptor ligation using the KAPA Hyperprep kit following manufacturer's instructions, except that 1.25 μM of Illumina-compatible indexed adapters (Pentabase) were ligated to A-tailed DNA for 60 min at 20°C and cleaned-up with Agencourt AMPure XP beads. For qChOR-seq experiments, indexed DNA from all time points in the same time course were then mixed together. Then, Click-IT was performed on 200 ng of indexed and mixed DNA for 30 min at room temperature under the following conditions (modified from Presolski et al., 2011): 1X Click-IT buffer (Click-iT EdU Alexa Fluor 488 Imaging Kit, Thermo Fisher Scientific), 0.5 mM biotin-TEG-azide (Berry & Associates), 0.1 mM $CuSO_4$, 0.5 mM THPTA (Sigma-Aldrich), 5 mM aminoguanidine (Sigma-Aldrich), and 10 mM sodium ascorbate. DNA fragments between 200-700 bp were size-selected using Agencourt AMPure XP beads and resuspended in TE. Next, to capture biotinylated products, MyOne Streptavidin T1 beads (Thermo Fisher Scientific) were washed three times with 1X B&W buffer (5 mM Tris-HCl pH 7.5, 0.5 mM EDTA, 1 M NaCl, 0.05% (V/V) Tween-20) and resuspended in 2X B&W buffer at a volume equal to the volume of biotinylated DNA. Streptavidin beads were then mixed with biotinylated DNA and rotated for 30 min at room temperature. Beads containing biotinylated DNA were washed four times with 1X B&W buffer, twice with 1X TE with 0.05% (V/V) Tween 20, and once with 10 mM Tris-HCl pH 7.5. Finally, beads were resuspended in double distilled water. PCR amplification of ChOR-seq samples was performed following the KAPA Hyperprep kit protocol using the streptavidin bead suspension as a template (10-15 cycles of PCR). Following PCR, streptavidin beads were purified using a magnetic rack, and the supernatant was cleaned-up with Agencourt AMPure XP beads.

In ChOR-seq experiments with biotin-dUTP performed without exogenous *Drosophila* chromatin, 120-240 μg of HeLa S3 chromatin was used per condition and streptavidin pull-downs were done on chromatin instead of on purified DNA. After the last wash with TE during ChIP, chromatin was separated from Protein A agarose beads by an incubation with elution buffer (10 mM Tris-HCl, 1 mM EDTA, 2% SDS, 15 mM DTT, supplemented with leupeptin, aprotinin, pepstatin, and PMSF; pH 8) for 30 min at 37°C in rotation. The supernatant was 1:20 with RIPA buffer and chromatin was then incubated with MyOne Streptavidin T1 beads to perform streptavidin pull-downs as described in Kim et al. (2009) and Kulyyassov et al. (2011). Briefly, MyOne streptavidin T1 beads were blocked by incubating with 1 μg/ml free biotin (Sigma) for 10 min at room temperature. Beads were then washed three times with RIPA buffer and 30 μg of input chromatin was pre-cleared with blocked beads for 1 hr at 4°C. After pre-clearing, supernatant was incubated with new, non-blocked T1 streptavidin beads overnight at 4°C. Beads were then washed for 5 min rotating at room temperature, once with RIPA buffer, twice with 2% SDS, once with RIPA 0.5 M NaCl, once with 1X LiCl buffer and twice with TE buffer. Chromatin was then purified and de-crosslinked as described for ChIP. End repair, A-tailing, size selection (200-700 bp) and library amplifications were done with 2 ng of DNA as starting material using the NEB Next Ultra DNA Library prep kit (New England Biolabs) following manufacturer's instructions.

Data Sequencing and Processing

ChOR-seq, ChIP-seq, replicated DNA and input samples from two independent time course experiments for each histone mark were sequenced at the Danish High-throughput DNA Sequencing Centre (https://seqcenter.ku.dk) and at the Biotech Research and Innovation Centre (BRIC) (https://www.bric.ku.dk) using Illumina HighSeq 4000 and NextSeq 500 machines to obtain 50 bp and 75 bp single-end reads, respectively. Reads were aligned to the February 2009 human genome assembly (GRCh37/hg19) and the April 2006 *D. melanogaster* genome assembly (BDGP R5/dm3) by Bowtie (Langmead et al., 2009) using parameters -m1–best. PCR duplicates were removed and uniquely mapped reads were extended to 250 bp (H3K4me3, H3K36me3, H3K79me3) or 500bp (H3K27me3) to account for the average library size. Replicated DNA was extended to 250 bp in early S time courses and to 500 bp in mid S time courses. Reads were then summed in 25 bp (H3K4me3, H3K36me3, H3K79me3, early S replicated DNA) or 500 bp (H3K27me3, H3, mid S replicated DNA) non-overlapping bins, unless otherwise specified, and reads were normalized to reads per million (RPM). Mapping and subsequent analysis of the data were done using a local Galaxy server and custom R scripts. In ChOR-seq experiments with exogenous DNA, human H3K4me3, H3K36me3, H3K79me3, H3K27me3 and histone H3 reads were divided by total *Drosophila* unique mapped reads to get quantitative information in the form of reference-adjusted RPM (RRPM) as described in Orlando et al. (2014). When comparing occupancy patterns between parental and nascent samples RPM or RRPM values were normalized to percentage of maximum. This normalization most appropriately represents the data, since in the parental sample we purify marks from the whole genome, while in nascent we only obtain signal from EdU-labeled domains, creating differences in signal:noise ratios that render direct comparisons inappropriate.

PTM Distribution Analyses

All data in figures correspond to replicate 1 of H3K4me3, H3K36me3, H3K79me3 and H3K27me3, respectively, unless otherwise specified. Since the ChOR-seq method purifies newly replicated chromatin, only bins overlapping with replicated regions in each experiment were considered for analysis. For H3K4me3, H3K36me3, and H3K79me3, peak calling was performed with MACS (Zhang et al., 2008) standard parameters and INPUT as control file. For H3K27me3, peak calling was performed with MACS "broad domain" parameters using replicate-matched parental ChIP-seq of histone H3 as a control. H3K4me3, H3K36me3, H3K79me3 and H3K27me3 domains were defined using parental ChIP-seq signal. Replicated regions were defined using MACS default "broad domain" parameters and replicate-matched parental ChIP-seq of histone H3 as a control and filtered for regions with a q-value greater than or equal to 0.05. Parental peaks were subsetted into 25 bp windows for H3K4me3, H3K36me3, and H3K79me3 and 500 bp windows for H3K27me3, and only windows that overlapped with replicated regions were included in downstream analyses. To calculate the mean difference in peak localization at individual loci, we selected H3K4me3 parental and nascent peaks overlapping only once and computed the absolute distance in bp between overlapping peaks at both ends.

Hilbert plots were created using Hilbertvis software (Anders, 2009). Average profiles and heatmaps were generated using Seqplot (Stempor and Ahringer, 2016). For H3K4me3 analyses, average profiles were centered on TSSs. For H3K27me3 analyses, average profiles were centered at the borders of H3K27me3 domains. Since H3K27me3 domain borders often overlapped with the borders of replicated DNA regions, we only included H3K27me3 borders that were at least 5 kb from the border of a replicated region in all analyses. For H3K36me3 and H3K79me3, average profiles were centered over open reading frames. Signal inside the open reading frame was normalized using the "anchored point" parameter in Seqplot to correct for gene length differences. When indicated, signal from average profiles were normalized using z-score $\left(z = \frac{x-\mu}{\sigma}\right)$ to focus on distribution differences, where μ is the mean of the population and σ the standard deviation.

Restoration Categories and Analyses

To study H3K4me3 restoration reads were summed in 500bp bins that overlapped with both parental H3K4me3 peaks and replicated regions. Regions were then classified into R0, R1, R6 and R12 categories according the time they need to reach T12 H3K4me3 levels. Only bins with R12/R(X) ratios greater than 1.5-fold and present in both replicates were considered for analysis. CpG islands overlapping R1 and R6 regions were respectively assigned to that region for analysis; CpG islands overlapping both a R1 and a R6 region were discarded. To ensure the CpG densities, calculated for coordinates in the hg18 genome assembly, remained accurate, R1 and R6 region coordinates were converted from hg19 to hg18 using UCSC LiftOver before defining overlaps.

To study the restoration dynamics of H3K27me3, reads were summed in 2 kb bins that overlapped with both parental H3K27me3 peaks and replicated regions. Regions were then classified into R0, R4, R10 and R24 categories according the time they need to reach T24 H3K27me3 levels. Only bins with R24/R(X) ratios greater than 1.5-fold and present in both replicates were considered for analysis.

For analysis of the H3K27me3 loss rate in the presence of EZH2 inhibitor, H3K27me3 loss categories were defined as the 2 kb windows present in replicated H3K27me3 parental peaks that showed a T0/T24 fold change smaller than 1.5 (low), between 1.5 and 3 (moderate) and bigger than 3 (high). Only bins that fit the criteria in the two independent replicates were considered for the analysis.

Cell Cycle Analyses

For analysis of cell cycle progression, synchronized cells were fixed with 70% ethanol and labeled with propidium iodide (10 μg/mL) for 30 min in the dark, before analysis on a FACSCalibur machine. FACS profiles were analyzed by FlowJo 10.0.8 software.

QUANTIFICATION AND STATISTICAL ANALYSIS

The statistical tests applied in this study are stated in the figure legends and were calculated using custom R scripts. In Figure S1E, r-values correspond to Pearson correlation. In boxplots, the bottom and top of boxes indicate the 25th and 75th percentiles, respectively, and middle lines indicate medians. Whiskers indicate the lowest and highest data points within 1.5 × interquartile range from the box.

DATA AND SOFTWARE AVAILABILITY

Replication timing was obtained from Repli-seq datasets GEO: GSM923449 (ENCODE Project Consortium, 2012). ChIP-seq of asynchronous HeLa cells for H3K27me3 (GEO: GSM733696), H3K4me3 (GEO: GSM733682), and EZH2 (GEO: GSM1003520) were taken from Bernstein et al. (2005). Asynchronous HeLa H3.3 ChIP-seq (GEO: GSM788633) was obtained from Ray-Gallet et al. (2011). Expression levels of genes were obtained from RNA-seq (GEO: GSM958735) (Mortazavi et al., 2008). Positions of TSSs were taken from the table of TSSs identified in Mortazavi et al. (2008). Exons, introns, 5′UTRs and 3′UTRs were defined using RefSeq annotations. CpG densities for CpG islands associated with promoters were taken from Illingworth et al. (2010). All original data generated in this study were deposited at GEO: GSE110354.

Molecular Cell, Volume *72*

Supplemental Information

Accurate Recycling of Parental Histones Reproduces the Histone Modification Landscape during DNA Replication

Nazaret Reverón-Gómez, Cristina González-Aguilera, Kathleen R. Stewart-Morgan, Nataliya Petryk, Valentin Flury, Simona Graziano, Jens Vilstrup Johansen, Janus Schou Jakobsen, Constance Alabert, and Anja Groth

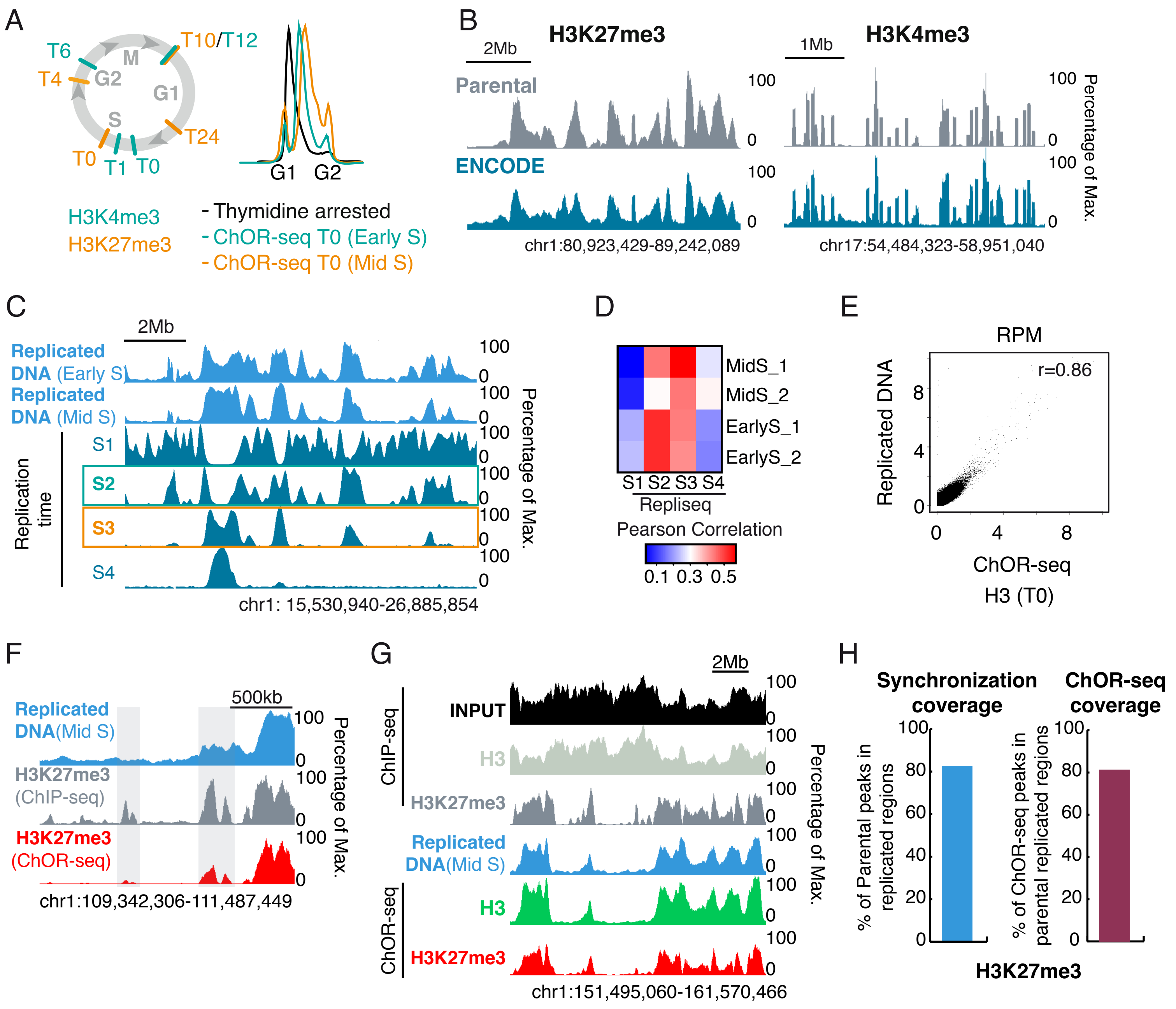

Figure S1. Tracking histone PTM occupancy after DNA replication with ChOR-seq, Related to Figure 1. (A) Outline of time course analysis for H3K4me3 and H3K27me3 ChOR-seq. (left) Experimental set-up. For H3K4me3 (green), cells were pulsed with EdU in early S phase and ChOR-seq samples were harvested immediately (T0, nascent) and 1 (T1), 6 (T6) and 12 (T12) hours later. For H3K27me3 (orange), cells were pulsed with EdU in mid S phase and ChOR-seq samples were harvested immediately (T0, nascent) and 4 (T4), 10 (T10) and 24 (T24) hours later. Cell cycle progression as indicated in the experimental set-up was assessed by FACS analysis of DNA content, as illustrated for T0 (right). **(B)** Profiles of parental H3K27me3 (left) and H3K4me3 (right) ChIP-seq in S phase synchronized cultures (gray) compared to ENCODE profiles from asynchronous Hela S3 cells (blue). Signal is scaled as percentage of maximum at the locus depicted. **(C)** Replicated DNA profiles from samples labelled in early or mid S phase compared with replication timing profiles determined by Repli-seq (ENCODE Project Consortium, 2012). Signal is scaled as percentage of maximum at the locus depicted. **(D)** Heat maps showing the global Pearson correlation between replicated DNA profiles of samples labelled in early or mid S and replication timing profiles determined by Repli-seq (ENCODE Project Consortium, 2012). Correlations are calculated from 500 bp non-overlapping windows and RPM. **(E)** Dot plots comparing signal in replicated DNA and pan-H3 nascent ChOR-seq. Dots represent 500 bp non-overlapping windows within replicated regions. R-values shown are Pearson correlations. **(F)** Profiles of replicated DNA (blue), parental H3K27me3 ChIP-seq (gray) and nascent H3K27me3 ChOR-seq (red). The left highlighted region shows a locus with parental signal but no nascent signal, owing to a low degree of replication (low enrichment of replicated DNA). The right highlighted region shows a locus with high parental signal but moderate nascent signal, owing to it being replicated to a moderate degree. The locus also includes a highly replicated region showing a strong agreement of the nascent signal with the parental. Signal is scaled as percentage of maximum at the locus depicted. **(G)** Parental ChIP-seq and nascent ChOR-seq profiles of pan-H3 and H3K27me3 using biotin-dUTP labelling and omitting spike-in chromatin. Replicated DNA profile is shown in blue. Signal was scaled as percentage of maximum at the locus depicted. **(H)** Bar plots showing the synchronization coverage (left) and ChOR-seq coverage (right) in H3K27me3 datasets from (G). Percentage is calculated from peaks subsetted into 500 bp non-overlapping windows.

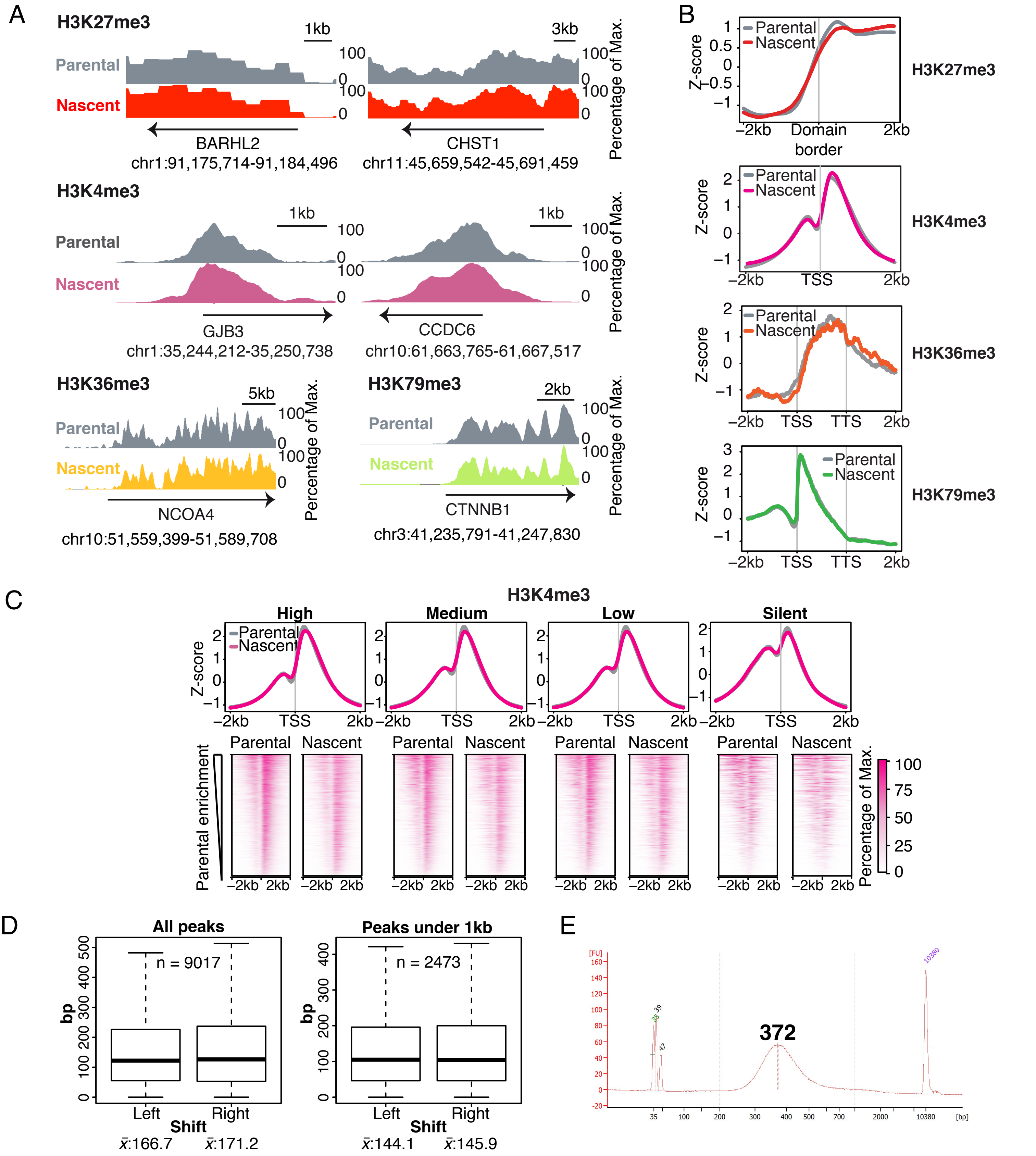

Figure S2. The histone H3 modification landscape is accurately reproduced in active and repressed genomic loci, Related to Figure 2. (A) Profiles of parental ChIP-seqs (gray and nascent H3K27me3 (red, H3K4me3 (pink, H3K36me3 (yellow, and H3K79me3 (green qChOR-seq at high resolution. Signal was scaled as percentage of maximum at the locus depicted. **(B)** Average profiles of parental and nascent H3K27me3, H3K4me3, H3K36me3, and H3K79me3 in replicate 2. H3K27me3 signal is plotted across 4 kb centered on borders of replicated H3K27me3 domains. H3K4me3 signal is plotted across 4 kb centered on replicated TSSs. H3K36me3 and H3K79me3 signals are plotted from 2 kb upstream to 2 kb downstream of replicated open reading frames. All data shown is z-score normalized. **(C)** Average profiles of parental and nascent H3K4me3 at TSSs parsed by expression level. Signal is plotted across 4 kb centered on replicated TSSs. All data shown is z-score normalized. **(D)** Box plots of difference in localization of parental and nascent H3K4me3 peaks (left and right shift) in base pairs for all peaks (left panel) and narrow peaks of less than 1 Kb (right panel). The narrow peaks containing a maximum of 5 nucleosomes provide the best resolution with respect to diffusion. n indicates the number of peaks analyzed. x̄ indicates the mean value of the absolute distance in bp between parental and nascent H3K4me3 peaks, note that the median is shown in the box plot. **(E)** Representative Bioanalyzer profile of a H3K4me3 ChOR-seq library. Taking into account the length of adaptors (approximately 120 bp), the average fragment size is 250 bp.

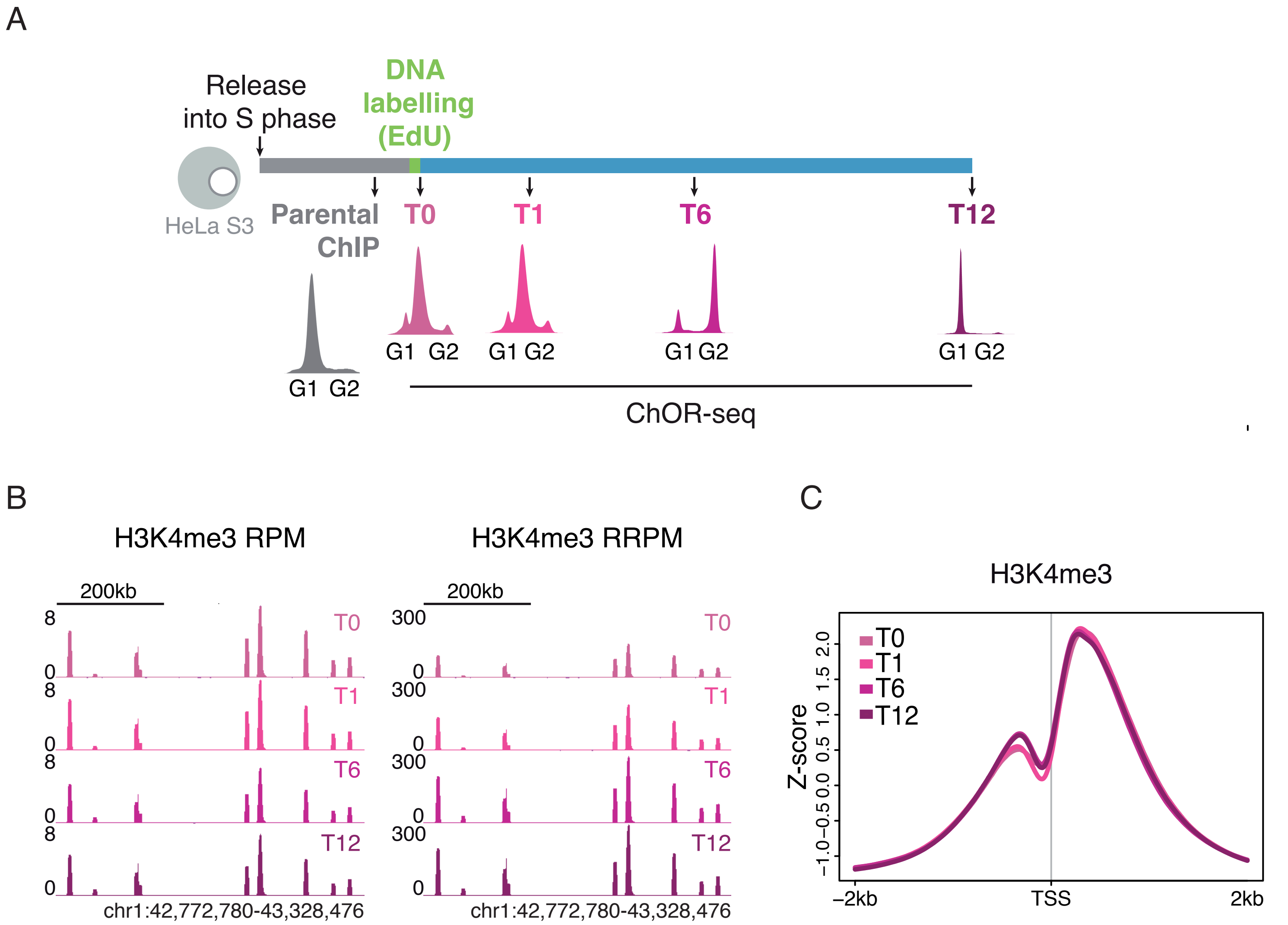

Figure S3. H3K4me3 restoration is complete within 6 hours and fastest in highly expressed promoters, Related to Figure 3. (A) Experimental set-up for H3K4me3 qChOR-seq. Parental chromatin was collected 1 hour before EdU labelling (10 minutes) and samples for qChOR-seq were harvested at the indicated time points post-EdU label. Cell cycle progression was assessed by FACS analysis of DNA content. **(B)** Comparison of H3K4me3 qChOR-seq profiles normalized to reads per million (RPM, left) or reference-adjusted reads per million using exogenous spike-in chromatin (RRPM, right). **(C)** Average profiles of H3K4me3 qChOR-seq. H3K4me3 signal is plotted across 4 kb centered on replicated TSSs. All data shown is z-score normalized.

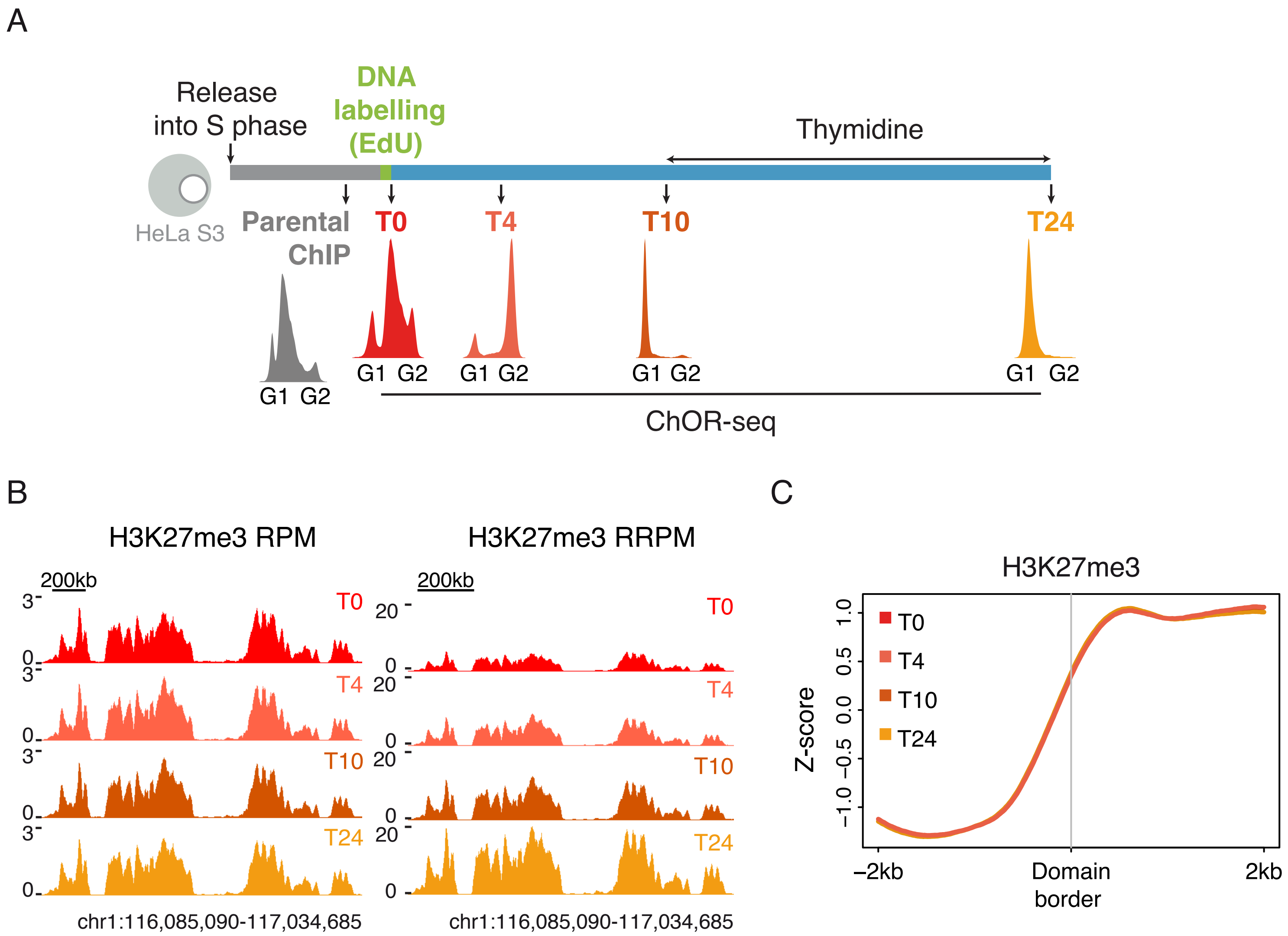

Figure S4. High PRC2 occupancy sites show faster H3K27me3 restoration, Related to Figure 4. (A) Experimental set-up for H3K27me3 qChOR-seq. Parental chromatin was collected 1 hour before EdU labelling (20 minutes) and samples for qChOR-seq were harvested at the indicated time points post-EdU label. Thymidine was added at 12 hours to prevent cells from entering a new round of replication. Cell cycle progression was assessed by FACS analysis of DNA content. **(B)** Comparison of H3K27me3 qChOR-seq profiles normalized to reads per million (RPM, left) or reference-adjusted reads per million using exogenous spike-in chromatin (RRPM, right). **(C)** Average profiles of H3K27me3 qChOR-seq. H3K27me3 signal is plotted across 4 kb centered on borders of replicated H3K27me3 domains. All data shown is z-score normalized.

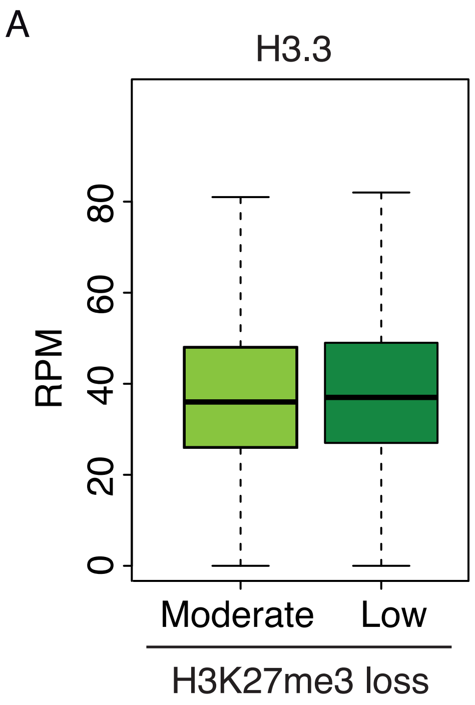

Figure S5. Parental H3K27me3 domains are stable across the cell cycle, Related to Figure 5. (A) Box plots of H3.3 ChIP-seq signal in moderate and low H3K27me3 loss regions. Data is subsetted into 2 kb non-overlapping windows and quantitated using reads per million (RPM). H3.3 data are from (Ray-Gallet et al., 2011).
